# Catabolism of serine enantiomers represses enterohemorrhagic *Escherichia coli* virulence factors via modulation of the nitrogen stress response

**DOI:** 10.1073/pnas.2532916123

**Published:** 2026-03-18

**Authors:** Emily Addington, Kabo R. Wale, Emily Horsburgh, Margot Fargeas, Leonidas Spathis, Weronika Leśniak, Saoirse Flavin, Patricia T. Rimbi, David R. Mark, Sofia Sandalli, Ester Serrano, Gavin Blackburn, Clément Regnault, Phillip D. Whitfield, James P. R. Connolly, Andrew J. Roe, Nicky O’Boyle

**Affiliations:** ^a^School of Infection and Immunity, University of Glasgow, Glasgow G12 8TA, United Kingdom; ^b^Department of Biological Sciences, University of Botswana, Gaborone 0000, Botswana; ^c^Department of Microbiology, School of Genetics and Microbiology, Moyne Institute of Preventive Medicine, Trinity College Dublin, Dublin D02 A2H0, Ireland; ^d^MVLS Shared Research Facilities, University of Glasgow, Glasgow G12 8QQ, United Kingdom; ^e^Newcastle University Biosciences Institute, Newcastle University, Newcastle-upon-Tyne NE2 4HH, United Kingdom

**Keywords:** *Escherichia coli*, nitrogen metabolism, EHEC, type 3 secretion system, virulence

## Abstract

Attaching and effacing pathogens, including enterohemorrhagic *Escherichia coli* (EHEC), colonize their preferred intestinal niche by sensing diverse host-, diet-, and microbiota-derived signals and coordinating the expression of virulence factors. D-serine, a host metabolite abundant in urine but scarce in the intestine, restricts EHEC colonization by transcriptionally repressing the type 3 secretion system (T3SS) while activating the SOS stress response. However, the mechanism underlying virulence regulation by D-serine remains unestablished. Here, we show that multiple amino acids, including L-serine converge on this pathway, repressing the T3SS without inducing the SOS response. Transcriptomic analyses showed a common response to D- and L-serine dominated by repression of nitrogen stress response genes. Mutational analysis identified the response regulators NtrC and Nac as essential mediators of T3SS repression by both serine enantiomers. Disruption of L-serine deaminase enzymes crucially revealed that T3SS repression depends on cytoplasmic ammonia/ammonium release rather than sensing of intact serine. While EHEC lacks canonical D-serine catabolic capacity, through metabolomics we provide evidence of oxidative deamination activity, capable of producing this regulatory signal. Together, these findings establish a mechanistic link between amino acid catabolism, nitrogen stress signaling, and virulence regulation in EHEC, highlighting how metabolic flux fine-tunes pathogen adaptation to intestinal niches.

## Significance

To optimize within-host fitness, bacterial pathogens exploit intricate sensory mechanisms to precisely modulate gene expression in response to host-associated cues. D-serine, a host-produced metabolite enriched in urine inhibits the type 3 secretion system (T3SS) of enterohemorrhagic *Escherichia coli* (EHEC). However, the molecular mechanism linking D-serine exposure to virulence repression remains incomplete. Here, we show that multiple amino acids, including L-serine, converge on this same regulatory pathway and repress the T3SS. A combination of transcriptomics, metabolomics, and targeted deletions reveal that this regulation is mediated by the release of ammonia, the nitrogenous product of serine breakdown, rather than by sensing of intact serine. Our data suggest that distal intestinal colonization by EHEC is facilitated by adaptation to amino acid-depleted environments.

The ability to sense and respond to environmental cues is an essential aspect of bacterial virulence. Attaching and effacing (A/E) pathogens use the locus of enterocyte effacement (LEE)-encoded type 3 secretion system (T3SS) to inject a suite of effector proteins into infected enterocytes (1). These hijack cellular processes, enabling the formation of protruding lesions on the host cell surface upon which the bacteria replicate. The LEE is divided into five polycistronic operons, *LEE1*—*LEE5*. Critical factors encoded in this island include the locus of enterocyte effacement regulator, Ler and the effector protein known as translocated intimin receptor, Tir. Ler functions as a master regulator of the system, binding to the promoters of all five LEE operons and activating their transcription (2, 3). Tir plays a key role in A/E lesion formation, with pairs of Tir molecules localizing to the enterocyte surface after translocation into the host cytosol where they engage in binding with pairs of intimin adhesins on the bacterial cell surface, enabling intimate epithelial colonization (4). As *ler* and *tir* are the first genes encoded in *LEE1* and *LEE5* respectively, their promoters are often used as reporters of T3SS activity. The activity of the LEE T3SS must be carefully regulated to ensure colonization of a favorable host niche. Enterohemorrhagic *Escherichia coli* (EHEC) is a classically gut-restricted pathotype of *E. coli*, distinct from extraintestinal pathogenic *E. coli* (ExPEC) that disseminate from the gut and cause infections at extraintestinal sites such as the bladder (5). EHEC strains have evolved a multitude of signal transduction mechanisms that regulate the LEE to enable precise induction or repression of T3SS-mediated tissue attachment in response to environmental change.

To facilitate the appropriate response to environmental cues, bacteria exploit various sensory mechanisms. For example, two component systems enable modulation of transcription in response to external stimuli. These comprise an inner membrane-localized sensor kinase and a cytoplasmic DNA-binding transcription factor that acts as the response regulator. Ligands bind the membrane bound histidine kinase, inducing autophosphorylation on a conserved histidine residue with the phosphoryl group subsequently being transferred to a conserved aspartate on the response regulator to modulate its activity (6). EHEC utilizes a vast array of sensory mechanisms to monitor its environment and appropriately control virulence. These include notable two component systems that detect host, dietary, and microbiota-derived signals [e.g., DcuSR, FusKR, QseC/KdpE, and EvgSA (7–10)]. Cytoplasmic transcription factors responsive to intracellular metabolites are also prevalent, for example ArgR which facilitates L-arginine sensing (11). Through these systems, EHEC fine-tunes virulence gene expression in response to niche-specific chemical cues.

D-serine is the chiral enantiomer of L-serine that plays a role in neurotransmission and is among the most abundant excreted urinary amino acids in humans (12). This metabolite is toxic to bacteria that cannot catabolize it and has been shown to elicit a dramatic global transcriptional response in EHEC defined by down regulation of the LEE T3SS and concurrent activation of an SOS-like stress response (5, 13). The potency of this transcriptional response is largely due to an evolutionary adaptation seen in ~95% of LEE-carrying *E. coli* where the gene encoding a transcriptional activator of D-serine catabolism, *dsdC* is lost (5). By contrast, 85% of pyelonephritis-associated uropathogenic *E. coli* (UPEC) (14) and 97.5% of neonatal meningitis-associated *E. coli* (NMEC) encode *dsdC* (15). The result of this EHEC adaptation is an inability to upregulate D-serine deaminase encoded by *dsdA* when subjected to exogenous D-serine. While the *dsdA* allele remains intact in EHEC isolates, they are unable to detoxify D-serine when present at levels found in urine (13, 14). Conversely, UPEC and NMEC isolates can effectively utilize D-serine as a source of carbon and nitrogen. The correlation between possession of *dsdC* and the host niche tropisms of these discrete pathotypes has led us to propose that D-serine acts as a regulator of pathogenic *E. coli* niche specificity (5). Importantly, L-serine is also among the most abundant urinary amino acids (12). While L-serine has been shown to promote fitness in mouse models of adherent and invasive *E. coli* and *Citrobacter rodentium* infection (16, 17), little is known about how L- and D-serine differ in their effects on global transcription or virulence regulation in EHEC. Our previous study on the effects of D-serine adaptation identified dependency on transporters CycA and SstT (18), analogous to the role of ArtMPQ in the L-arginine response (11). However, given the absence of a canonical D-serine response regulator such as DsdC in EHEC, uncovering the precise mechanism by which D-serine represses the T3SS has proven difficult.

In addition to precisely coordinating the expression of virulence and fitness factors within the host, pathogens must navigate a variety of stress-inducing environmental conditions. Such stresses stem from the host immune response, competing microbiota, and the physicochemical parameters (pH, osmotic stress, nutritional gradients) of the host environment. Nitrogenous compounds are key nutrient sources within the host (16, 17) with nitrogen starvation inducing a sophisticated signal transduction pathway known as the nitrogen stress (Ntr) response (19). This response enables a reprogramming of global gene expression to allow scavenging and fixation of nitrogen from the environment. Glutamate and glutamine act as the dominant cellular nitrogen donors accepting nitrogen from deaminated amino acids, free ammonium, and other amine donors and distributing this nitrogen to meet cellular demand for biomolecule synthesis (20). Low glutamine levels are the primary trigger for activation of the Ntr response with the system being most extensively studied by culturing *E. coli* K-12 in minimal medium with limiting concentrations of ammonium (21). Upon sensing of low glutamine, a uridylation and phosphorylation cascade is activated, culminating in phosphorylation of NtrC (21), a transcription factor with a direct regulon of 49 genes that includes amino acid transporters, amino acid biosynthetic enzymes, and an additional transcription factor known as the nitrogen assimilation control factor, Nac (22). A central activity of the Ntr response is the activation of glutamine synthase, encoded by *glnA* which acts to fix ammonia by transferring an amine group to glutamate, generating glutamine (19). Through the activities of NtrC and Nac, approximately 40% of the *E. coli* genome can be modulated to facilitate adaptation to low nitrogen niches (21). Beyond modulation of metabolism, it is also becoming increasingly apparent that the Ntr system contributes to virulence in important human pathogens including *Shigella flexneri*, *Pseudomonas aeruginosa*, and *E. coli* (23–26).

In this study, we identify significant overlap between the global effects of D- and L-serine, with repression of Ntr response genes being common to both amino acids. Using genetics, transcriptome analyses, metabolomic profiling, and in vitro virulence assays we further dissect mechanistic aspects of the effects of these amino acids on cellular physiology and virulence regulation in EHEC.

## Results

### Several Amino Acids Repress the EHEC T3SS, Whereas SOS Induction is D-Serine-Specific.

**Fig. 1.**
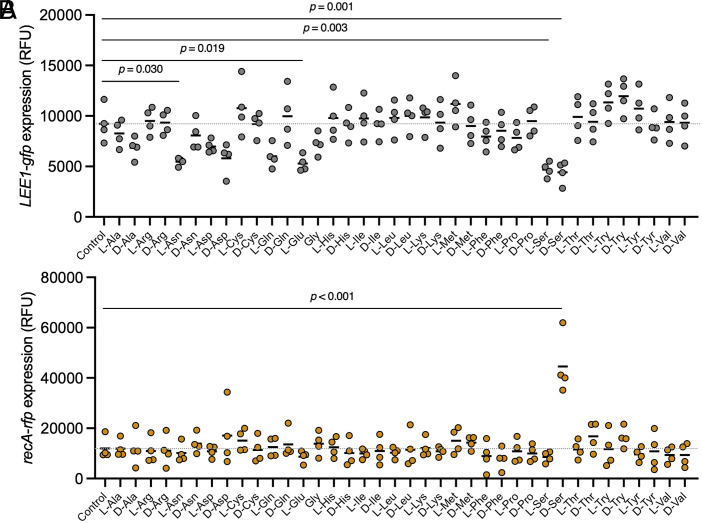
Repression of the LEE T3SS occurs with several amino acids, while SOS induction is highly specific to D-ser. TUV93-0 (p*LEE1-gfp+recA-rfp*) was cultured in MEM for 5 h in microtiter plates with or without inclusion of 1 mM of each indicated amino acid. Fluorescence intensity at 485_ex_/520_em_ (*A*), 544_ex_/620_em_ (*B*), and absorbance (OD_600 nm_) were recorded using a BMG Fluostar Optima plate reader. Normalized Relative Fluorescence Units (RFU) are reported for 4 replicate experiments with means indicated by black lines. Statistical analysis was conducted using Ordinary One-way ANOVA with Dunnett’s test for multiple comparisons. Complete statistics for treatments found to be not significant are included in the source data (Dataset S4).

Given that D-serine can repress the LEE T3SS—a property with potential therapeutic relevance—we sought to determine whether other amino acids exert similar effects. We also reasoned that identifying redundant responses might provide insight into D-serine’s mechanism of action. We first screened all 19 natural chiral amino acids and the achiral amino acid glycine for ability to modulate the activities of the *LEE1* and *recA* promoters (*LEE1p* and *recAp*) using a dual eGFP/RFP fluorescence-based reporter plasmid (18). Amino acids were tested at a concentration of one millimolar which is equivalent to the maximal concentration of D- and L-serine found in the urine (5, 27). D-glutamate and the known toxic amino acid L-selenocysteine (28) severely inhibited growth and were therefore excluded from the dataset (*SI Appendix*, Fig. S1). In addition to D-serine, L-asparagine, L-glutamate, and L-serine significantly reduced *LEE1* promoter activity (Fig. 1*A*). By contrast, induction of *recA* was exclusive to D-serine (Fig. 1*B*). The dose response to each enantiomer of serine was similar for *LEE1p* repression with no inhibition being observed at concentrations of 0.2 mM and lower (*SI Appendix*, Fig. S2*A*). This indicated that while there is redundancy in the T3SS response to amino acids, there are aspects of the response to D-serine that are unique.

### Comparison of Global Response to D- and L-Serine Reveals Parallel Repression of the Nitrogen Stress Response.

**Fig. 2.**
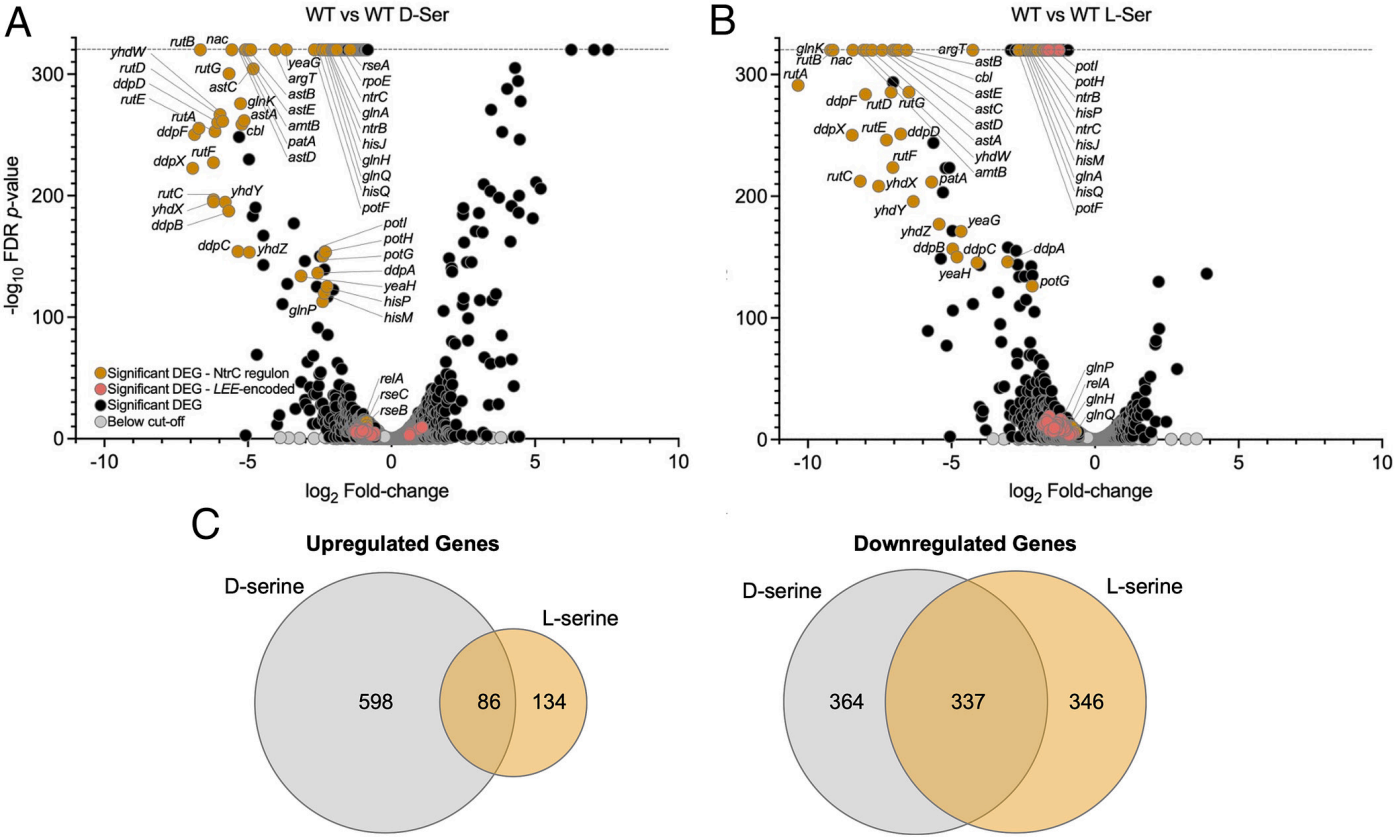
The NtrC-regulon dominates overlap between genes repressed by L- and D-serine. TUV93-0 was cultured to late exponential phase in MEM with and without 1 mM D- or L-serine before collecting samples for RNA-seq. Volcano plots were constructed by comparing triplicate biological replicates of the control condition (without addition of amino acid) to 1 mM D-ser (*A*) or 1 mM L-ser (*B*). Data displayed are log_2_-adjusted fold-changes and -log_10_-FDR-adjusted *P*-values. Differentially expressed genes (DEGs) having a *P*-value of 0 were arbitrarily assigned a below threshold of quantitation -log_10_-FDR-adjusted *P*-value of 320 (indicated by the dotted line). Orange dots indicate significant DEGs that belong to the NtrC regulon (RegulonDB); pink dots indicate genes encoded within the *LEE*; black dots indicate significantly affected genes outside the LEE and the known NtrC regulon; gray dots indicate genes not reaching the cut-off of ≥1.5-fold induction/repression or *P* ≤ 0.05. (*C*) Venn diagrams illustrating the overlap in up and downregulated genes affected by D- and L-serine. Source data are provided (Dataset S4).

Since L-serine could repress the *LEE1p* without activating *recA,* we speculated that the global response to chiral enantiomers of serine would be markedly distinct. Furthermore, while D-serine exerts a modest inhibition of growth in MEM-HEPES (*SI Appendix*, Fig. S2 *B* and *C*), L-serine increases growth rate and results in a higher stationary phase optical density, indicative of increased biomass accumulation (*SI Appendix*, Fig. S2*D*), highlighting a general distinction in physiological response to the chiral enantiomers. To gain further insight, RNA-seq analysis was performed on EHEC in the presence and absence of 1 mM D- or L-serine. Differential expression analysis indicated that 701 genes were downregulated in response to D-serine (Fig. 2 *A* and *C* and Dataset S1), 683 genes were downregulated in response to L-serine (Fig. 2 *B* and *C*), and 337 of these genes were commonly downregulated in response to both enantiomers. Among the genes commonly downregulated by D- and L-serine were the majority of the genes encoded within the LEE (Fig. 2 and *SI Appendix*, Figs. S3 and S4*A*) and several non-LEE-encoded effector (NLE) genes (*SI Appendix*, Fig. S4B). By contrast, 684 genes were upregulated in response to D-serine, 220 genes were upregulated in response to L-serine (Fig. 2 *B* and *C*), and 86 of these genes were commonly upregulated in response to both enantiomers. Therefore, despite the contrasting effects of D- and L-serine on growth dynamics, there is considerable overlap in their effects on global transcription. Most strikingly, 18 out of the 20 most downregulated genes in both conditions belonged to the 49 gene NtrC regulon (22). D-serine reduced expression of the entire 49 gene regulon, while L-serine reduced expression of 45 (Dataset S1). In summary, while there are important physiological differences between the effects of D- and L-serine, there is a surprising level of similarity in their effects on global transcription in T3SS-inducing media, and this is dominated by repression of genes involved in the nitrogen stress response. This indicates that the nitrogen stress response is active in T3SS-inducing media and relief of nitrogen stress by D- and L-serine correlates with T3SS repression.

### Characterizing the Role of NtrC in the Regulation of the D- and L-Serine T3SS Response.

While D-serine is known to repress the EHEC T3SS (5), a complete regulatory mechanism has yet to be established. It has recently been reported that L-glutamine can repress the EHEC T3SS in MEM-HEPES via interference with NtrC mediated activation of the *LEE1p* binding transcription factor PchA (25). Together with our observations of a shared ability of D- and L-serine to reduce expression of the Ntr regulon and the LEE T3SS, this was highly suggestive of a role for NtrC in T3SS repression by D- and L-serine. We therefore analyzed the response to D- and L-serine in a mutant lacking *ntrC*.

**Fig. 3.**
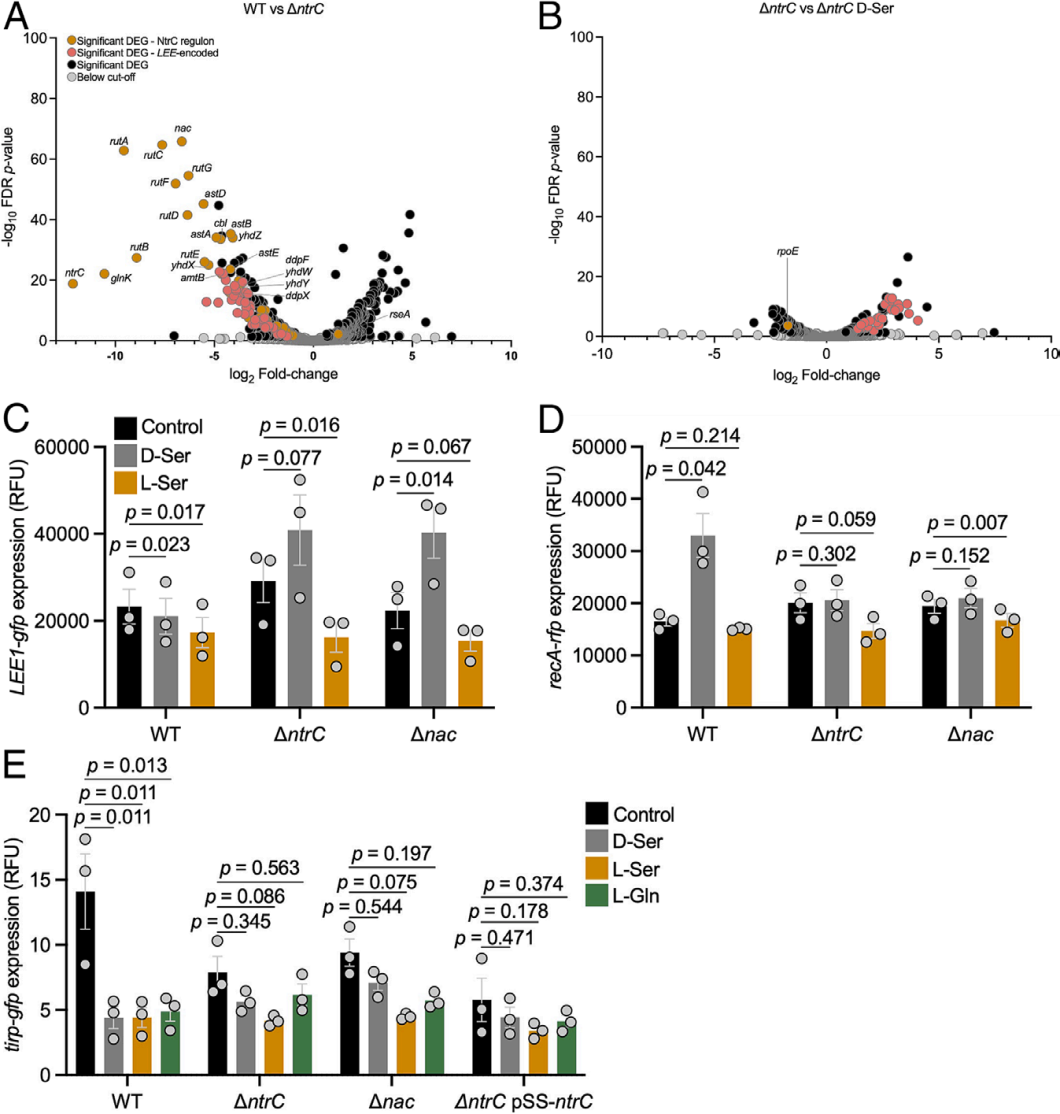
Transcriptional regulators of the Ntr stress response are required for T3SS repression and SOS activation by D-serine. (*A* and *B*) Global analysis of the effect of *ntrC* deletion and the response of an *ntrC* deletion mutant to D-serine. Volcano plots display log_2_-adjusted fold-changes and −log_10_-FDR-adjusted *P*-values. Orange dots indicate significant DEGs that belong to the NtrC regulon (RegulonDB); pink dots indicate genes encoded within the *LEE*; black dots indicate significantly affected genes outside the LEE and the known NtrC regulon; gray dots indicate genes not reaching the cut-off of ≥1.5-fold induction/repression, or *P* ≤ 0.05. (*C*–*E*) Reporter assays validating the results of global transcriptional analysis. TUV93-0 (p*LEE1-gfp+recA-rfp*) was cultured in MEM for 5 h with or without inclusion of 1 mM of each indicated amino acid. Fluorescence intensity [FI: 485_ex_/520_em_ (**C* and *E**) and 544_ex_/620_em_ (*D*)] and absorbance (OD_600 nm_) were recorded. Normalized RFU are reported for three replicate experiments with SE of the means (SEM) indicated by error bars. Statistical analysis was conducted using two-tailed Student’s *t*test. Source data are provided (Dataset S4).

Deletion of *ntrC* resulted in 375 upregulated genes and 425 downregulated genes (Fig. 3*A*). In agreement with its role as an activator of the nitrogen stress response, 36 genes of the direct Ntr regulon were downregulated by deletion of *ntrC*. Only *rseA* was upregulated in response to deletion of *ntrC*. The mutant also exhibited a modest growth impairment likely stemming from impaired ability to adapt to the nitrogen limiting conditions in T3SS-inducing media (*SI Appendix*, Fig. S5). Surprisingly, supplementation of the growth medium with D-serine improved growth of the Δ*ntrC* mutant, again suggesting that EHEC is not fundamentally incapable of utilizing D-serine. In terms of its effect on T3SS regulation, disruption of *ntrC* largely phenocopied supplementation with D- or L-serine with strong downregulation of the LEE and certain NLEs. Those most significantly affected by D- and L-serine included *nleA* and *espR3* (*SI Appendix*, Fig. S4).

We next compared the transcriptome of the *ntrC* mutant in the presence and absence of D-serine in attempts to uncover unique aspects of the response to D-serine (Fig. 3*B*). The magnitude of the global response to D-serine was strikingly reduced in the absence of *ntrC*. Only 120 genes were significantly upregulated, and 85 genes downregulated in response to D-serine in the Δ*ntrC* mutant. Most notably, a mutant lacking *ntrC* failed to alter the expression of the Ntr regulon (with the exception of *rpoE*) and failed to repress the genes for the T3SS (Fig. 3 and *SI Appendix*, Fig. S4). In fact, 30 of 41 *LEE*-encoded genes were upregulated by D-serine in this strain. No upregulation of *recA* or genes belonging to the LexA SOS regulon was observed for the Δ*ntrC* mutant in response to D-serine.

To validate the transcriptome data, a series of reporter experiments were performed. As the Ntr response is facilitated both through direct regulation by the DNA binding transcription factor NtrC (49 gene regulon) and through indirect regulation via Nac (553 gene regulon) the *LEE1/recA* dual reporter was tested in deletion mutants lacking either *ntrC* or *nac* (22). The reporter also allowed us to easily compare the effects of D-serine with L-serine and other amino acids. Consistent with our transcriptome analysis, no repression of *LEE1p* by D-serine was observed in mutants lacking *ntrC* or *nac*, while repression of *LEE1p* was maintained with L-serine (Fig. 3*C*). Similarly, D-serine failed to induce *recAp* activity in these deletion mutant backgrounds (Fig. 3*D*). While not significant in the Δ*ntrC* mutant, *LEE1p* appeared to be modestly induced by D-serine in these mutant backgrounds. After consulting the wild type transcriptome analysis (*SI Appendix*, Fig. S4), it was seen that at the transcript level, *LEE1* did not follow the rest of the *LEE* with respect to directionality of altered expression by D-serine. D-serine was found to induce *ler* with a modest fold change of 1.92 (*P* = 1.25 × 10^−8^). To more robustly ascertain transcriptional effects of these amino acids on the T3SS, a *tir* promoter (*tirp*) reporter was also tested. Strong repression of the *tir* promoter was observed (Fig. 3*D*) with D-serine, L-serine, and the previously characterized T3SS-repressing amino acid L-glutamine (25). Disruption of *ntrC* and *nac* led to reduced *tir* expression at the promoter level consistent with the results for global profiling of the Δ*ntrC* mutant (Fig. 3*A*). While a modest reduction in *tirp* activity was observed with D-serine, L-serine, and L-glutamine in all mutant backgrounds, this was not found to be significant, therefore we conclude that NtrC and Nac are required for effective repression of the LEE T3SS by D-serine and L-serine.

Fang *et al.* (2023) showed that the NtrC-dependent repressive effects of L-glutamine were mediated by the LrhA-Pch regulatory pathway (25). This pathway facilitates *LEE* activation via induction of the *LEE1*-binding transcription factors PchA and PchB by LrhA (*SI Appendix*, Fig. S6*A*). We therefore constructed a mutant lacking *lrhA* and analyzed the effects of D- and L-serine on *LEE1p* activity. As expected, deletion of *lrhA* led to a reduction in *LEE1p* activity (*SI Appendix*, Fig. S6), however, inclusion of D- or L-serine resulted in further repression of *LEE1p* indicating that additional regulators beyond the LrhA-Pch network mediate the repressive effects of D- and L-serine on the *LEE1* promoter.

### Amino Acid Deamination Provides a Source of Ammonium to Repress the EHEC T3SS.

**Fig. 4.**
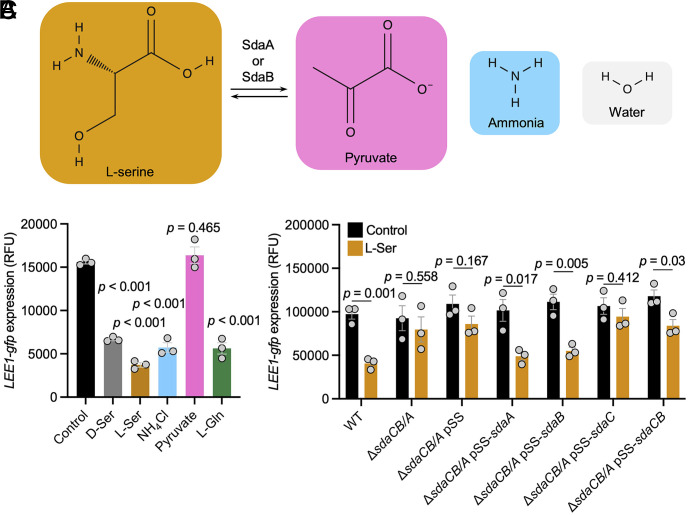
Breakdown of L-serine via serine dehydratase I and II is required for repression of the LEE T3SS. (*A*) Schematic illustration depicting the activities of L-serine dehydratase I (SdaA) and L-serine dehydratase II (SdaB) and associated products of catabolism. (*B*) TUV93-0 (p*LEE1-gfp+recA-rfp*) was cultured in MEM for 4 h with or without inclusion of 1 mM of each indicated amino acid or breakdown product. Fluorescence intensity (FI: 485_ex_/520_em_) was recorded on a BMG Fluostar Optima plate reader. (*C*) TUV93-0 (p*LEE1-gfp+recA-rfp*) and indicated mutants were cultured in MEM for 5 h with or without inclusion of 1 mM L-serine. Fluorescence intensity (FI: 485_ex_/520_em_) was recorded on a Biotek Synergy HT plate reader. (*B* and *C*) Normalized RFU are reported for three replicate experiments with SE of the means (SEM) indicated by error bars. Statistical analysis was conducted using two-tailed Student’s *t*test. Source data are provided (Dataset S4).

As both D- and L-serine relieved the nitrogen stress response in MEM-HEPES, we speculated that catabolism, specifically deamination, of both amino acids had occurred leading to satisfaction of cellular nitrogen demand. The breakdown products of L-serine deamination via L-serine dehydratase I (SdaA), L-serine dehydratase II (SdaB), and the anaerobically active L-serine dehydratase III (TdcG) are pyruvate, ammonia, and water (reaction summarized, Fig. 4*A*). In solutions of neutral or acidic pH ammonia becomes efficiently protonated to yield ammonium. To determine if intact amino acids, or either breakdown product (ammonia or pyruvate) was responsible for T3SS repression, we compared *LEE1p* activity in the presence of equimolar concentrations of pyruvate, ammonium chloride, and the amino acids previously shown to repress *LEE1p* (Fig. 4*B*). Pyruvate has been shown to induce the *LEE1p* as cells enter stationary phase (29). In our log-phase assay, pyruvate had no discernible effect on *LEE1p* activity, however ammonium chloride repressed the *LEE1p* to a similar extent as D-serine, L-serine, and L-glutamine.

To confirm that intracellular deamination of L-serine facilitated T3SS repression, we deleted the L-serine dehydratase enzymes encoded by *sdaA* and *sdaB* together with the L-serine transporter encoding gene *sdaC*, encoded in the same operon as *sdaB*. The triple mutant displayed no repression of *LEE1p* in the presence of 1 mM L-serine, while the wild type displayed 2.39-fold reduced *LEE1p* activity (*P* = 0.001) (Fig. 4*C*). A series of XylS/Pm benzoic acid inducible complementation vectors were also tested without addition of inducer. Leaky expression of the system facilitated restoration of T3SS repression only when either *sdaA* or *sdaB* was present (Fig. 4*C*). No restoration of T3SS repression was seen with empty pSS or pSS-*sdaC*. These results confirm that liberation of ammonium from exogenously added L-serine is required for T3SS repression.

While catabolism of L-serine via SdaA/SdaB would be expected, the case with D-serine is less clear. Due to evolutionary loss of *dsdC*, the transcription factor necessary for induction of D-serine deaminase (DsdA), EHEC isolates fail to productively detoxify D-serine. An alternative enzyme responsible for D-serine deamination in EHEC has not yet been described. Nevertheless, the data presented here thus far are highly suggestive of a yet unrecognized mechanism of D-serine deamination.

**Fig. 5.**
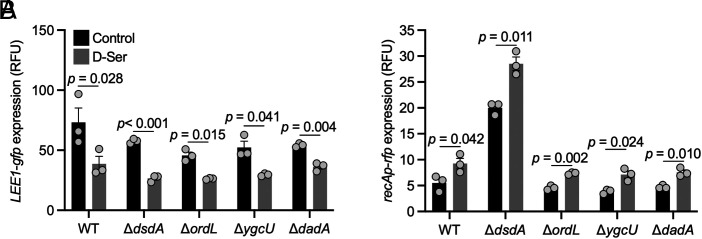
The T3SS-repressing and SOS-inducing effects of D-serine are not elicited exclusively through DsdA, OrdL, YgcU, or DadA. (*A* and *B*) TUV93-0 (p*LEE1-gfp+recA-rfp*) was cultured in MEM for 5 h with or without inclusion of 1 mM D-serine. Fluorescence intensity (FI: 485_ex_/520_em_ (*A*), 544_ex_/620_em_ (*B*), and absorbance (OD_600 nm_) were recorded on a VarioSkan lux plate reader. Normalized RFU are reported for three replicate experiments with SE of the means (SEM) indicated by error bars. Statistical analysis was conducted using two-tailed Student’s *t*test. Source data are provided (Dataset S4).

We first hypothesized that DsdA may be expressed at a low constitutive level sufficient for relief of the Ntr response upon addition of exogenous D-serine but insufficient to alleviate D-serine-associated stress responses. Disruption of *dsdA* failed to restore T3SS activity in the presence of D-serine excluding DsdA as a primary driver of D-serine deamination (Fig. 5*A*). Surprisingly, disruption of *dsdA* resulted in heightened *recAp* activity in the absence of D-serine (Fig. 5*B*), indicating a role for DsdA in reducing basal SOS response levels in minimal medium. Second, the 426 amino acid gamma-glutamyl putrescine oxidoreductase encoded by *ordL* was found to be 2.23-fold induced by D-serine (*P* = 4.22 × 10^−10^). Of note, D^29^-L^381^ of OrdL comprises Pfam domain PF01266 (FAD-dependent oxidoreductase), a domain shared with R^2^-L^329^ of the 347 amino acid *Homo sapiens* D-amino acid oxidase (DAO). Despite its potential role in D-amino acid metabolism and its induction by D-serine, disruption of *ordL* did not abolish D-serine–mediated repression of the T3SS. An additional FAD-binding oxidoreductase encoded by *ygcU*/*z4084* was also found to be 1.69-fold induced by D-serine (*P* = 0.03). Again, deletion of this gene failed to restore T3SS activity in the presence of D-serine. The final potential D-serine deaminase investigated was DadA, a D-amino acid dehydrogenase involved in peptidoglycan turnover. This enzyme also possesses the Pfam domain PF01266 (spanning R^2^-L^397^ of DadA) seen in *Homo sapiens* DAO and OrdL, facilitating oxidative dehydrogenation of D-alanine, D-methionine and D-phenylalanine, D-serine, D-proline and D-threonine (30). Despite this, D-serine effectively repressed *LEE1p* in the *dadA* mutant. Collectively, these data indicate that the putative deaminases tested are not solely responsible for deamination-dependent T3SS repression in response to D-serine.

### Distinct Alterations in the Cellular Metabolome of D-Serine and L-Serine Treated EHEC.

Having observed that enzymatic deamination was required for the T3SS repressing effects of L-serine (Fig. 4), and that D-serine could relieve the Ntr response in a similar manner to L-serine, we conducted a series of metabolomics experiments to confirm our speculation that D-serine was indeed broken down via deamination. Direct quantitation of ammonia presents a challenge. First, it is rapidly converted to ammonium at neutral pH, with ammonium in turn being rapidly fluxed into the cellular transamination network via glutamine synthase (20), the expression of which is induced by NtrC (31). Second, the low mass of ammonium and volatility of ammonia prohibit chromatographic resolution via LC-MS. For these reasons, we conducted untargeted LC-MS with the aim of identifying either the oxidative carboxylic acid deamination product β-hydroxypyruvate or the dehydration carboxylic acid product pyruvate. Given that D-serine exerts a diversity of alterations in global transcription distinct from L-serine, including activation of the SOS response, we speculated that oxidative deamination may occur.

**Fig. 6.**
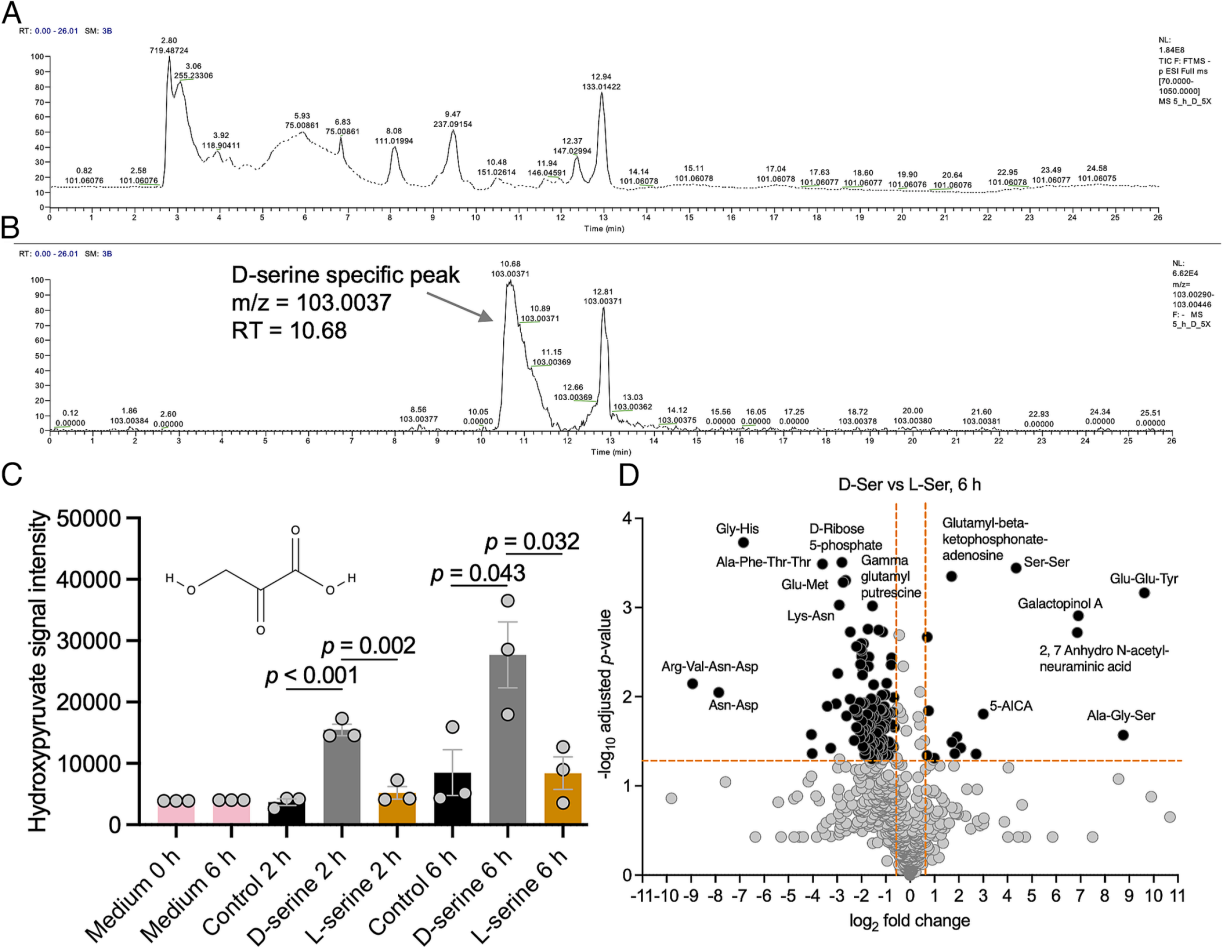
Chiral enantiomers of serine induce distinct alterations in the EHEC metabolome including D-serine-specific accumulation of the oxidative breakdown product hydroxypyruvate. (*A* and *B*) LC-MS data derived from TUV93-0 following 5 h of exposure to D-serine show a peak of m/z 103.0037 at RT 10.68. (*C*) Targeted quantitation of hydroxypyruvate following 2 and 6 h of growth in control MEM-HEPES, or MEM-HEPES with 1 mM of each amino acid demonstrates D-serine specific accumulation. Signal intensities are reported for 3 biological replicates with SE of the means (SEM) indicated by error bars. Statistical analysis was conducted using two-tailed Student’s *t*test. (*D*) Untargeted metabolomics demonstrates distinct metabolome profiles following 6 h exposure to 1 mM D- and L-serine. Source data are provided (Dataset S4).

Populations of EHEC were cultured for 2, 2.5, 3, 3.5, 4, 5, and 6 h in the presence and absence of 1 mM L- or D-serine before normalizing cell density, extracting cellular metabolites and subjecting to LC-MS. We also extracted metabolites from the 5 h populations using a 5× cell density preparation (5 × 10^9^ cells per extraction) in case the lower cell density compromised resolution. Data were interrogated using the IDEOM pipeline (32) (Dataset S2). As shown in the mass spectrum and chromatogram (Fig. 6 *A* and *B*), the 5× D-serine extraction showed a peak of mass to charge ratio (m/z) of 103.003 and retention time (RT) 10.68. This peak was absent in the extraction from a 5× preparation derived from cells incubated without D-serine (*SI Appendix*, Fig. S7). Extractions from 6 h cultures at the lower cell density showed a reduced intensity m/z 103.003 peak in the D-serine-treated population (*SI Appendix*, Fig. S8) but not in the control (*SI Appendix*, Fig. S9) or L-serine-treated population (*SI Appendix*, Fig. S10). The detected m/z of 103.003 was consistent with β-hydroxypyruvate which is isomeric with malonate.

To accurately resolve the identity of the detected peak, and to perform relative quantitation of additional metabolites, a repeat experiment was conducted with triplicate high cell density extractions being performed on 2 and 6 h cultures with parallel analysis of 1 mM malonate and 1 mM β-hydroxypyruvate standards. In addition, MEM-HEPES medium following 0 and 6 h of incubation at 37 °C was also tested. The IDEOM pipeline putatively identified 802 metabolites (Dataset S3). Analysis of malonate and β-hydroxypyruvate standards demonstrated clear chromatographic resolution with a RT of 13.14 and 10.90 respectively (*SI Appendix*, Fig. S11). This targeted approach allowed us to categorically demonstrate significant D-serine-specific accumulation of β-hydroxypyruvate at both 2 and 6 h (Fig. 6*C*). By contrast, the levels of pyruvate detected at 2 h were similar in the presence and absence of D-serine, while at 6 h a significant reduction in pyruvate was observed comparing D- and L-serine treatment (*SI Appendix*, Fig. S14*A*). This leads us to conclude that D-serine is likely to be catabolized via oxidative deamination, a reaction which yields ammonia, β-hydroxypyruvate, and hydrogen peroxide. Importantly, neither oxidation-specific breakdown product (β-hydroxypyruvate, nor hydrogen peroxide) was capable of modulating the *LEE1* or *recA* promoters at a 1 mM concentration, the maximum potential yield from 1 mM D-serine (*SI Appendix*, Fig. S12) indicating that these transcriptional responses are not solely due to the products of oxidative catabolism.

Beyond direct products of serine degradation, untargeted analysis yielded several additional insights. The Kyoto Encyclopaedia of Genes and Genomes (KEGG) pathways most significantly upregulated by D-serine (relative to L-serine) were galactose metabolism and glycine, serine and threonine metabolism, while the most significantly downregulated were the citrate cycle and glyoxylate metabolism (*SI Appendix*, Fig. S13). Many peptides were found to be differentially abundant comparing D- and L-serine treated cells at 6 h, perhaps indicative of changes in the global proteome (Fig. 6*D*). These included D-serine specific enrichment of Glu-Glu-Tyr and Ala-Gly-Ser with depletion of Gly-His, Ala-Phe-Thr-Thr, and Arg-Val-Asn-Asp. Of particular note was the D-serine-specific accumulation of Ala-Ser which was just beyond the significance cut-off at 6 h but significant at 2 h (*SI Appendix*, Fig. S14*B*). Incorporation of exogenous D-amino acids into peptidoglycan has been demonstrated in several phylogenetically distinct bacteria (33, 34). This most often occurs at the fourth position of the muropeptide stem, replacing D-alanine. It is therefore unsurprising that a concomitant decrease in Ala-Ala was observed with exposure to D-serine (*SI Appendix*, Fig. S14*C*). D-serine-specific accumulation of Ser-Ser was also detected (Fig. 6*D* and *SI Appendix*, Fig. S14*D*) which could be indicative of muropeptide alteration at both positions 4 and 5, however a mechanism for how this might occur is unclear. Also of note, the modified sialic acid 2,7 anhydro-alpha-N-acetyl neuraminic acid was completely undetectable in all control media and cell extracts with the exception of the D-serine-treated 6 h sample (*SI Appendix*, Fig. S14*E*). Collectively, these data highlight that the cellular metabolome of EHEC is differentially shaped by chiral enantiomers of serine and that this includes the generation of the oxidative breakdown product β-hydroxypyruvate.

### Chiral Enantiomers of Serine Differ in Their Ability to Interfere With Attaching and Effacing Lesion Formation in vitro.

Transcriptional repression of the LEE T3SS by L-serine has not yet been reported. To ascertain whether these repressive effects at the transcript level could functionally interfere with the T3SS in an infection context, and to determine the role of NtrC in this process, a filamentous actin staining assay (FAS) was employed using HeLa cells and fluorescently labeled EHEC strains (carrying p*rpsM-gfp*). In tandem, HeLa cell infections were carried out with unlabeled bacteria, the cell layers were washed to remove nonadherent bacterial cells and the tissue layers were lysed with 1% (v/v) Triton X-100. Total numbers of bacteria were enumerated in the inoculum, pooled wash and supernatant, and lysate.

**Fig. 7.**
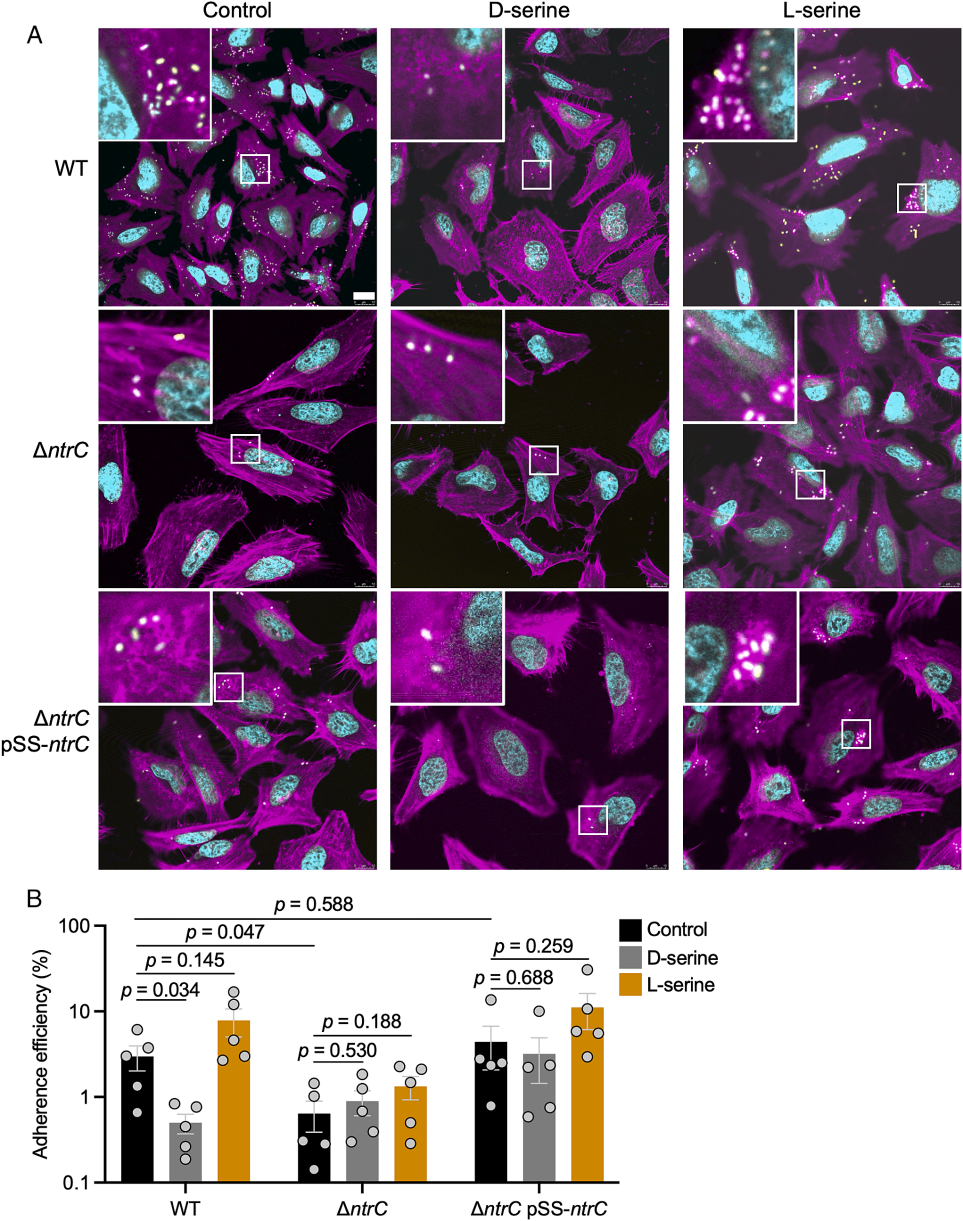
Chiral enantiomers of serine display contrasting ability to interfere with attaching and effacing lesion formation in vitro. (*A*) HeLa cells were infected with TUV93-0 (p*rpsM-gfp*), Δ*ntrC* (p*rpsM-gfp*), and Δ*ntrC* (p*rpsM-gfp*/pSS-*ntrC*) for 3 h before washing, fixing, and staining to allow for qualitative assessment of lesion formation. Infections were carried out in MEM-HEPES without inclusion of FCS or L-glutamine. 1 mM D- or L-serine was included or omitted as indicated. Bacterial cells are pseudocolored yellow, actin pseudocolored magenta, and nuclei pseudocolored cyan. (*B*) Nonfluorescent variants of the above strains (without p*rpsM-gfp*) were used to infect HeLa as previously described. Following four PBS washes, PBS + 1% Triton X-100 was added and incubated for fifteen min to lyse the HeLa cells. The cells were scraped from the plate, mixed by repeatedly pipetting and serially diluted to titer adherent bacteria. Adherent CFU is expressed as a percentage of the inoculum added. Data from five replicate experiments are reported with SE of the means (SEM) indicated by error bars. Statistical analysis was conducted using two-tailed Student’s *t*test. Source data are provided (Dataset S4).

In agreement with our previous reports, inclusion of D-serine led to a marked reduction in A/E lesion formation by FAS assay (Fig. 7*A*). A reduction in cell-associated WT bacteria was also apparent upon D-serine inclusion (Fig. 7*B*). Surprisingly, despite the potent effects of L-serine in reducing *LEE1p* activity in pure culture (Figs. 1 and 3) and repressing LEE genes at the transcript level (Fig. 2 and *SI Appendix*, Fig. S3 and S4), no qualitative reduction in A/E lesion formation or significant alteration in cell-associated bacteria was observed with L-serine. Disruption of *ntrC* did lead to a significant reduction in A/E lesion formation (Fig. 7*A*) and adherent bacterial numbers (Fig. 7*B*), and no further reduction in these phenotypes was seen by addition of D-serine to the Δ*ntrC* mutant, demonstrating that NtrC is required for the inhibitory effects of D-serine on A/E lesion formation in vitro. Complementation of the deletion with the pSS-*ntrC* plasmid yielded a modest increase in overall adhesion, yet the repressive effect of D-serine was not restored in this strain. We hypothesized that while leaky expression of serine deaminase was sufficient for complementation (Fig. 4*C*) more robust induction was required to completely restore the activity of NtrC. We therefore repeated HeLa infections with the Δ*ntrC* pSS-*ntrC* strain, at varying concentrations of the inducer 3-Methylbenzoic acid (3-MeBzO). We observed that T3SS induction peaked at 20 μM 3-MeBzO and trended downward thereafter (*SI Appendix*, Fig. S15). Moreover, the repressive effects of D-serine on attachment to HeLa cells were restored by induction of *ntrC* with 20 μM 3-MeBzO confirming that the response to D-serine requires functional levels of NtrC expression.

We hypothesized that the apparent lack of ability of L-serine to reduce the numbers of cell-associated bacteria might stem from enhanced growth of EHEC in the presence of L-serine (*SI Appendix*, Fig. S2*B*). L-serine is a favored carbon and nitrogen source for *E. coli* and therefore its inclusion may have facilitated rapid growth and thus enhanced bacterial cell numbers at the 3 h sampling timepoint. We therefore quantitated the total bacterial numbers (adherent + pooled wash and supernatant) in each well. Inclusion of L-serine did not yield an increase in bacterial numbers per well in either the wild type or Δ*ntrC* mutant (*SI Appendix*, Fig. S16*D*). A significant increase was only seen with the complemented strain. This indicates that the high numbers of adherent CFU observed for L-serine compared with D-serine treatment do not stem from enhanced bacterial growth in the presence of L-serine. Interestingly, a significant decrease in overall numbers was observed for D-serine treatment with wild type EHEC. D-serine exerts only a modest effect on growth in pure culture (*SI Appendix*, Fig. S2 *B*–*D*). HeLa cells may therefore inhibit replication of EHEC in the presence of D-serine and this may, in addition to direct T3SS repression, contribute to the reduced numbers of A/E lesions and adherent bacteria observed with D-serine treatment.

## Discussion

Amino acids play dual roles during infection. They act both as important sources of carbon and nitrogen to fuel growth, and serve as niche-specific cues that regulate the expression of virulence factors. Assimilation of amino acids derived from dietary protein is generally considered to be completed in the small intestine (35). However, endogenous colonic sources of amino acids also exist, including mucin, shed epithelial cells, and products of microbial metabolism (35). Amino acids exist in spatial gradients and are dynamically shaped by microbiota catabolism (36), cross-feeding (37), as well as host diet and state of health (38). Furthermore, amino acids that exist in both L- and D-form (e.g., D-serine) are excreted in urine at millimolar concentrations (27, 39) and have been shown to modulate niche specificity of pathogens (5, 14, 40). Here, we show that several amino acids can repress the LEE T3SS and for L-serine this phenotype depends on enzymatic deamination. We propose that redundant amino acid sensing acts as a proximal vs distal intestinal niche specific cue, enabling EHEC to activate the T3SS specifically within its preferred colonic niche.

Despite the efficiency of intestinal assimilation, several studies highlight a role for dietary amino acids in modulating the outcome of enteric infection (16, 17, 41, 42). During colonization of streptomycin-treated mice, *E. coli* K-12 has been shown downregulate biosynthetic pathways for L-aspartate, L-glutamine, L-glutamate, and L-serine and to upregulate serine transporters DsdX and TdcC, and L-serine deaminases SdaA and TdcG (35). This suggests that during intestinal colonization, exogenous amino acids are favored over de novo biosynthesis. Furthermore, cecal contents from colitic mice promotes the growth of adherent and invasive *E. coli* and *C. rodentium* in a serine-deaminase-dependent manner, while cecal contents from noninflamed mice does not, suggesting that amino acid content of the gut can be shaped by inflammatory status (16). This confirms that L-serine is an important nutrient in the inflammatory niche. In a similar study, metabolism of L-serine, L-lysine, and L-asparagine was shown to provide *S. enterica* a competitive fitness advantage in an ex vivo cecal model (17). Importantly, these studies show that the contribution of amino acids to expansion within the inflammatory niche is driven by their availability within the cecum. Whether inflammation promotes availability of amino acids within EHECs preferred colonic niche remains to be determined. Moreover, it has been shown that the *C. rodentium* T3SS is not repressed by exogenous L-glutamine while the EHEC T3SS is (25). As such, distal niche sensing via depletion of amino acids may be specific to EHEC.

Although proximal assimilation of dietary amino acids would support a colonization-promoting colonic niche for EHEC, enteric ammonia has been reported to follow an inverse gradient. Ammonia generated through host metabolism is processed in the hepatocytes generating urea (43). Circulating urea can freely flow across the gut barrier where it is acted upon by urease positive gut microbiota to once again generate ammonia/ammonium that can be taken up and used as a nitrogen source by the microbiota, or expelled in the feces (44). The concentration of ammonia in distal colonic luminal contents of rats has been reported to range from 39 to 74 mM (45), while human stool ammonium ranges from 12 mmol kg^−1^ to 24 mmol kg^−1^ (46). Despite this abundance of nitrogen, there is evidence to suggest the Ntr response is important during enteric infection. For example, during infection of mice with *Klebsiella pneumoniae*, activation of ethanolamine metabolism via the NtrC–RpoN regulatory cascade is crucial for the pathogen to outcompete the resident gut microbiota (47). In another study, mutation of *ntrC* in *E. coli* K-12 led to a significant reduction in competitive fitness in streptomycin-treated mice (48). Another recent study using in vivo inducible Tn-seq showed that disruptions in *ntrC* have a significant fitness defect in the *C. rodentium* infection model (49). Interestingly, in addition to supporting survival in nitrogen-deplete conditions, NtrC has been shown to contribute to fitness in nitrogen rich conditions in vitro (31). While these studies illuminate a potentially critical role for NtrC during enteric infection, how diverse enteric nitrogenous cues are integrated to control virulence factor expression remains to be determined.

A key aim of this study was to develop insight into the mechanism underlying T3SS regulation by D-serine. We therefore hypothesized that a specific transcriptional regulator may mediate this response. We recently identified binding sites for DsdC outside of the *dsdCXA* loci in NMEC (50) and showed that transcriptomic responses of UPEC and NMEC to D-serine are distinct (13). However, EHEC lacks DsdC thus making the regulatory mechanism underlying T3SS regulation difficult to resolve. A D-serine responsive two component sensor GrpPQ that induces type 1 fimbriae was recently identified in UPEC (51) but this system is also absent in EHEC. Here, we report that D-serine relieves the Ntr response in EHEC resulting in downregulation of NtrC and the entire direct NtrC regulon. NtrC, together with its associated nitrogen response sigma factor, σ^N^ has been described as a positive regulator of the LEE T3SS in EHEC strain TW14359 (52). A complex, yet incomplete mechanism was proposed involving posttranscriptional inhibition of σ^S^, a factor known to repress the LEE T3SS (53). Mitra *et al.* (2012) speculated that inhibition of σ^S^ activity leads to production of an unknown activator of the Pch-Ler regulatory network. EHEC encodes five homologous Pch-like regulators, two of which (PchA and PchB) contribute to optimal LEE expression by directly activating the *LEE1*/*ler* promoter (54). Deletion of *pchA* and *pchB* which are dependent upon the upstream activator LrhA has been shown to phenocopy deletion of the LEE master regulator, *ler* (55). More recently, the repressive effect of L-glutamine on the LEE was shown to depend on both *ntrC* and the LrhA-Pch-Ler regulatory network (25). However, we found that disruption of LrhA does not overcome T3SS repression by D- or L-serine, suggesting that PchA and PchB are not the exclusive mediators of serine-dependent T3SS regulation. While a specific DNA-binding transcriptional regulator essential for mediating these responses has not been identified, the Ntr response regulators NtrC and Nac have been implicated. Importantly, the consistency of the T3SS regulatory phenotype in distinct nitrogen stress response regulator mutants (*ntrC* and *nac*) reinforces the proposed involvement of this pathway. The mechanism of regulation is likely to be indirect as neither regulator is known to occupy regulatory regions of the LEE directly. Future research should focus on transcription factors known to be regulated by Nac, as single deletions of both Nac and NtrC eliminated the effect of D- and L-serine on LEE genes, and deletion of NtrC greatly reduced the transcriptomic response to D-serine. It should also be noted that the Nac regulon includes the D-serine transporter CycA, which we previously showed was required for T3SS regulation and SOS induction by D-serine (18). As such, the phenotypic effects of deleting *ntrC* and *nac* may stem from impaired serine uptake as well as disrupted T3SS regulation.

Considering that L-serine could transcriptionally repress the LEE, it was surprising that it was incapable of reducing the formation of A/E lesions during infection of HeLa cells. The most likely explanation for this is competition between host and microbe for L-serine during infection. Infection experiments of 3 h duration were performed in MEM-HEPES without addition of FBS or L-glutamine to enable direct assessment of the effects of individual amino acids. It is likely that the resulting nitrogen limitation created a demand for nitrogen in both host and microbe resulting in less availability of serine for microbial uptake, deamination, and subsequent T3SS repression. In support of this, L-serine supplementation in HeLa infections did not promote increased microbial growth (*SI Appendix*, Fig. S16*D*), whereas in pure EHEC cultures, increased growth was observed (*SI Appendix*, Fig. S2*B*).

The reprogramming of EHEC gene expression by serine enantiomers is underpinned by distinct metabolic rewiring, highlighting the interconnection between metabolism and virulence. Satisfaction of cellular nitrogen demand and concurrent relief of the Ntr response by L-serine is perhaps unsurprising as EHEC encodes three distinct L-serine deaminase enzymes which break down L-serine into pyruvate and ammonia (16, 56). *E. coli* can subsequently use spontaneously ionized ammonia as a sole source of nitrogen through the activity of the Ntr-regulated enzyme glutamine synthase thus satisfying nitrogen demand (20, 48). To date, it has been reported that EHEC strains are incapable of utilizing D-serine due to loss of DsdC (5, 13, 14). Nevertheless, our observation that D-serine can relieve the Ntr response and liberate β-hydroxypyruvate in EHEC is suggestive of D-serine catabolism. A missing link here is the identification of a specific catabolic enzyme. While deletion of *dsdA*, *ordL*, *ygcU,* and *dadA* individually was insufficient to overcome T3SS repression by D-serine, it is plausible that two or more of these enzymes have redundant functionality. Given the inability of EHEC to use D-serine as a sole source of carbon (57), it is also plausible that the hypothesized D-serine catabolic enzyme is poorly expressed or weakly active in the conditions tested. In addition to acting as a coenzyme, pyridoxal phosphate has been shown to catalyze nonenzymatic breakdown of cysteine (58) and oxidative deamination of valine, leucine, isoleucine, and phenylalanine (59). Therefore, it is also possible that an enzyme independent mechanism of D-serine breakdown is at play.

There are several aspects of the physiological response of EHEC to D-serine that remain unsolved, including the mechanism of SOS induction. Our identification of oxidative catabolism activity led us to speculate that the breakdown product β-hydroxypyruvate or hydrogen peroxide might be responsible. Subinhibitory concentrations of hydrogen peroxide have indeed been shown to induce the SOS response in *E. coli*, however sensitivity to these effects is strain-dependent (60–62). In this study, neither breakdown product was seen to elicit *recAp* induction at a concentration consistent with maximal yield from D-serine. It is tempting to speculate that misincorporation into the cell wall in place of canonical D-amino acids might lead to an SOS-like response, and this is backed up by our observation of Ala-Ser accumulation under D-serine exposure. Indeed, cell wall targeting antibiotics including beta lactams are known to induce the SOS response (63). While the precise mechanism underlying SOS induction remains elusive, stress originating from cell wall perturbations is a plausible explanation for SOS activation independent of oxidative breakdown products.

In conclusion, this study has identified that the NtrC-regulated nitrogen stress response is essential for optimum T3SS gene expression in EHEC, crucially mediating the repressive effects of D- and L-serine on this system. We provide evidence of a vital role for deamination in the L-serine response and demonstrate that a distinct oxidative breakdown product accumulates under D-serine exposure. Despite significant overlap in the transcriptome of EHEC exposed to D- and L-serine, important nuances exist, including D-serine specific activation of the SOS response. These distinctions are mirrored in metabolomic analyses and highlight the importance of deep molecular characterization in resolving how microbes respond to chiral enantiomers of amino acids. Such insight will be crucial in order to ensure effective therapeutic application of specific amino acids for bacterial infections.

## Materials and Methods

A complete list of bacterial strains, plasmids, and oligonucleotides used in this study can be found in *SI Appendix*, Tables 1–3. A detailed description of all methodology is included in Supplementary *Materials and Methods*. This includes Bacterial strains and culture conditions, Screening of amino acids for T3SS repression and SOS induction, Promoter fusion reporter assays, Growth curves, Transcriptome analysis using RNA-seq, Gene disruption by lambda red recombineering, *Trans* complementation using the pSS-XylS/Pm system, Metabolite extraction, Analytical high-performance liquid chromatography coupled with mass spectrometry (LC-MS), FAS assay, Quantitative analysis of bacterial attachment, and Statistical analysis.

## Supplementary Material

Appendix 01 (PDF)

Dataset S01 (XLSX)

Dataset S02 (XLSX)

Dataset S03 (XLSX)

Dataset S04 (XLSX)

## Data Availability

RNA sequencing data have been deposited at The European Nucleotide Archive [PRJEB101298 (64) and PRJEB89299 (65)]. The empty pSS XylS/Pm vector sequence has been deposited at NCBI (PX119074) (66). Study data are included in the article and/or supporting information.
