# Supplementary material for: Catabolism of serine enantiomers represses enterohemorrhagic Escherichia coli virulence factors via modulation of the nitrogen stress response
Source: Proc Natl Acad Sci U S A. 2026 Mar 18;123(12):e2532916123. doi: 10.1073/pnas.2532916123 (PMC13012123; doi:10.1073/pnas.2532916123)
Supplement: Supplementary file 1 — Appendix 01 (PDF) [file pnas.2532916123.sapp.pdf]

Supplementary Information

Catabolism of serine enantiomers represses Enterohemorrhagic *Escherichia coli* virulence factors via modulation of the nitrogen stress response

Emily Addington, Kabo R Wale, Emily Horsburgh, Margot Fargeas, Leonidas Spathis, Weronika Leśniak, Saoirse Flavin, Patricia T Rimbi, David R Mark, Sofia Sandalli, Ester Serrano, Gavin Blackburn, Clément Regnault, Phillip D Whitfield, James PR Connolly, Andrew J Roe, and Nicky O'Boyle

Contents:

Supplementary Materials and Methods

Description of Supplementary Datasets S1-S4

Supplementary Figures S1-S16

Supplementary Table 1 – Bacterial strains used in this study

Supplementary Table 2 – Plasmids used in this study

Supplementary Table 3 – Oligonucleotides used in this study

Supplementary References

Supplementary Materials and Methods

Bacterial strains and culture conditions

Bacterial strains were routinely cultured overnight at 37°C, 200 rpm in lysogeny broth (LB, Miller's recipe – 10 g l^{-1} tryptone, 5 g l^{-1} yeast extract, and 10 g l^{-1} NaCl). For experiments, overnight LB cultures were diluted one hundred-fold into pre-warmed MEM-HEPES (Sigma Alrich, M7278) without inclusion of L-glutamine. Plasmids were retained by incorporation of antibiotics at the following concentrations: chloramphenicol, 25 µg ml^{-1}; ampicillin, 100 µg ml^{-1}; kanamycin 50 µg ml^{-1}; hygromycin, 200 µg ml^{-1}. A complete list of strains and plasmids used are provided in Supplementary Tables 1 and 2.

Screening of amino acids for T3SS repression and SOS induction

A dual fluorescence reporter system was used to analyse the effects of a panel of amino acids on promoter activity of *LEE1p* and *recAp*. Aqueous amino acid (except for tyrosine) standards were prepared at a 5 mM concentration and filtered through a 0.2 µm filter to sterilise. Inocula comprising MEM-HEPES, 30 µg ml^{-1} chloramphenicol and an eighty-fold dilution of TUV93-0 (p*LEE1p-gfp* + *recAp-rfp*), or TUV93-0 (pDUAL) promoterless vector control overnight culture were prepared. Inocula were added to amino acid standards, or water control in a 4:1 ratio to dilute amino acids to 1 mM. Due to poor solubility, D- and L-tyrosine, were dissolved directly in MEM-HEPES with 25 µg ml^{-1} chloramphenicol at a 1 mM concentration before filter sterilising. One-hundred-fold dilutions of overnight cultures of each strain were prepared directly in tyrosine-containing wells. Plates were incubated at 37°C, 200 rpm for four hours with $OD_{600\ nm}$, GFP fluorescence intensity ($485_{ex}/520_{em}$) and RFP fluorescence intensity ($544_{ex}/620_{em}$) being recorded on a BMG Fluostar Optima plate reader. Data are presented as RFU – background subtracted fluorescence intensity relative to $OD_{600\ nm}$.

Promoter fusion reporter assays

Reporter assays using EHEC and isogenic mutants were carried out using an adaptation of the microtitre plate-based screening experiment above. Overnight LB cultures of TUV93-0 (p*LEE1p-gfp* + *recAp-rfp*) and TUV93-0 (pDUAL) promoterless vector control were diluted one hundred-fold into polystyrene tubes containing pre-warmed MEM-HEPES without L-glutamine supplementation. Plasmids were selected for using 25 µg ml^{-1} chloramphenicol throughout. Where pSS XylS/Pm complementation plasmids were employed, 100 µg ml^{-1} ampicillin was also included. Tubes were incubated at 37°C, 200 rpm. At the indicated timepoints, samples were removed and transferred to a microtitre plate before recording $OD_{600\ nm}$, GFP fluorescence intensity ($485_{ex}/520_{em}$) and RFP fluorescence intensity ($544_{ex}/620_{em}$) on a BMG Fluostar Optima, Biotek Synergy HT, or Varioskan lux plate reader. Data are presented as RFU – background subtracted fluorescence intensity relative to $OD_{600\ nm}$.

Growth curves

Overnight LB cultures were diluted one hundred-fold into Erlenmeyer flasks containing pre-warmed MEM-HEPES. Flasks were incubated at 37°C, 200 rpm. Samples were taken at hourly intervals and transferred to cuvettes before measuring $OD_{600\ nm}$ using an Eppendorf Bio Spectrometer. Specific growth rate was calculated as previously reported[1].

Transcriptome analysis using RNA-seq

RNA sequencing (RNA-seq) was carried out as previously described[1]. Briefly, strains were incubated at 37°C, 200 rpm in MEM-HEPES to an optical density ($OD_{600\ nm}$) of 0.8 +/- 0.1. Approximately 10^9 cells were harvested and RNA extracted using Purelink RNA mini extraction kit (Thermo Fisher). Following treatment with Turbo DNase (Thermo Fisher), RNA samples were purified by phenol chloroform isoamyl alcohol extraction and assessed for

integrity by electrophoresis. Library preparation and sequencing was carried out by Glasgow Polyomics. Ribosomal RNA depletion was conducted with QiaSeq FastSelect 5S/16S/23S Kit (Qiagen). Approximately 15 million single end 75 bp reads were acquired per sample using NextSeq 500 (Illumina). Read quality was assessed using FASTQC (minimum Phred threshold of 20). Comparative transcriptomic analysis was conducted using the EDGER package within CLC Genomics Workbench (Qiagen). A statistical cut-off of false discovery rate (FDR)-adjusted $p < 0.05$ and an absolute fold change cut-off of +/- 1.5 was applied to the dataset. Raw data has been uploaded to the European Nucleotide Archive under the accession numbers: PRJEB101298 and PRJEB89299. To conduct pathway enrichment analysis, differentially expressed genes (absolute fold change ≥ 1.5 and corrected *p*-value threshold < 0.05) were separated into up- and down-regulated lists for each condition. The gene ontology enrichment analysis was performed using the CompareCluster function in the ClusterProfiler[2] R package. This method conducts over-representation analysis (ORA) based on gene counts (hypergeometric test), determining if the number of significant genes belonging to a specific GO category is greater than expected by chance. To ensure comprehensive annotation of virulence factors absent in standard databases, a hybrid annotation database was constructed by merging *Escherichia coli* K-12 GO terms (org.EcK12.eg.db) with manually curated terms for the Type III Secretion System (T3SS) derived from the EDL933 genome annotation. *P*-values were adjusted for multiple testing using the Benjamini-Hochberg method, and pathways with an adjusted *p*-value < 0.05 were considered significant.

Gene disruption by lambda red recombineering

Genes were inactivated using adaptations of the Datsenko and Wanner method[3]. All PCR fragments used for cloning and deletion were amplified using Q5 polymerase mix (NEB). A complete list of primers used is provided in Supplementary Table 3. A linear deletion cassette

for inactivation of *ntrC* was produced by amplification of the *cat* cassette on pKD3 using ntrC-red-F and ntrC-red-R. The resulting fragment comprising 50 bp regions homologous to the genomic DNA flanking *ntrC* was column purified using Qiaprep PCR purification kit (Qiagen). TUV93-0 was transformed with pSIM18 and selected on LB agar with 200 µg ml^{-1} hygromycin at 28°C overnight. Transformant colonies were grown to an optical density ($OD_{600\ nm}$) of 0.4 - 0.6 before inducing the red system by incubating at 42°C for 20 min. Cells were collected, washed three times with water and electroporated with one microgram of the linear *ntrC* deletion cassette. Following two hours recovery at 37°C in super optimal complete medium, mutants were selected by plating on LB with 25 µg ml^{-1} chloramphenicol. Generation of TUV93-0 mutants using 50 bp homology regions was found to have low efficiency. We therefore modified the approach to assemble deletion cassettes with larger 500-700 bp homology regions. For example, 500 bp of DNA upstream of *nac* was amplified with oligos Nac-DelA-184 and Nac-DelB-pKD, 500 bp of DNA downstream of *nac* was amplified with oligos Nac-DelC-pKD and Nac-DelD-p184, the pKD3 cassette was amplified with oligos pKD-F and pKD-R, and the pACYC184 vector backbone was linearised by amplification with pACYC184-lin-Fw and pACYC184-lin-Rev. The vector backbone was gel extracted and purified while other fragments were directly column purified using Qiaprep Spin Gel and PCR Purification Kit (Qiagen). Homology regions on each of the fragments facilitated a four-fragment assembly using Hifi Assembly Mix (NEB) according to manufacturer's instructions. The assembled construct was transformed by heat shock into *E. coli* DH5α before recovery, and selection on LB with 25 µg ml^{-1} chloramphenicol. Correct vector assembly was confirmed by colony PCR using primers pACYC-seq-For2 and pACYC.check.R. Product from the confirmed colony was diluted 1,000-fold and used as template in eight separate PCR reactions. Products were pooled before column purification. One microgram of purified product was used to transform lambda red-induced pSIM18 transformants as outlined above. For all mutants, recombination of the desired resistance cassette was confirmed by PCR using check

primers with annealing sites outside of the region of recombination, followed by Sanger sequencing of products. Resistance markers were eliminated by FLP recombination using pCP20.

Trans **complementation using the pSS-XylS/Pm system**

A construct (pSS-XylS/Pm, hereby referred to as pSS) comprising the benzoic acid inducible Pm promoter and *xylS* from pSEVA238[4], and the ColE1 origin, beta lactamase and copy number enhancer *rop* from pBR322[5] was made by gene synthesis (Twist Bioscience). The empty vector sequence has been deposited in the NCBI database (Accession: PX119074). The vector was linearised by amplification with primers pSS-lin-F and pSS-lin-R. Product was gel extracted and assembled with the required gene(s). For example, the coding sequence of *sdaA* was amplified using primers sdaA-pSS-F and sdaA-pSS-R. The product was assessed by electrophoresis before mixing with purified pSS-lin product and assembling vector and insert using Hifi assembly mix (NEB) according to manufacturer's instructions. The reaction mixture was transformed into *E. coli* DH5α before recovery, and selection on LB with 100 µg ml^{-1} ampicillin. Clones with the correct assembly were identified by PCR before isolating plasmids using Qiaprep Spin miniprep kit (Qiagen) and subjecting to Sanger sequencing. Sequence confirmed plasmids were electroporated into the relevant mutant, for example Δ*sdaA,* and selected on LB with 100 µg ml^{-1} ampicillin. Where necessary, p*LEE1-gfp+recA-rfp* or p*rpsM-gfp*, and the required complementation plasmid were sequentially transformed into the same strain, in which case final selection took place on LB with ampicillin and chloramphenicol. Leaky expression of the pSS-borne XylS/Pm promoter facilitated complementation of some phenotypes, others required addition of the inducer 3-methyl benzoic acid (3-MeBzO).

Metabolite extraction

Overnight cultures of TUV93-0 were diluted one hundred-fold in pre-warmed MEM-HEPES without L-glutamine supplementation. Cultures were incubated at 37°C, 200 rpm for the indicated duration. At each time point, optical density was recorded, and a volume equivalent to 10^9 or 5×10^9 cells was taken and chilled to 4°C by briefly placing in ethanol and dry ice. The cells were placed on water ice before being pelleted by centrifugation at 10,000 × *g* for two minutes. Cell pellets were washed with ice cold MS grade water before snap-freezing in ethanol and dry ice and storing at -70°C. To extract metabolites, each cell pellet was resuspended in ice cold MS grade chloroform, methanol and water in a 1:3:1 ratio. A blank extraction was also performed. Tubes were vortexed at 4°C for one hour. Extracts were placed on ice before centrifuging at 13,000 × *g* for three minutes and transferring supernatants to fresh pre-chilled tubes on dry ice. A pooled quality control sample was also prepared by combining aliquots from each individual sample. Extracts were stored at -70°C.

Analytical high performance liquid chromatography coupled with mass spectrometry (LC-MS)

Ten microlitres of each sample was run on a polymeric hydrophilic interaction chromatography ZIC-pHILIC column (150 mm × 4.6 mm, 5 µm, Sequant) using the Dionex UltiMate 3000 system (Thermo Fisher). A linear gradient of 20 mM ammonium carbonate (A) and acetonitrile (B) was used over a period of 26 minutes as follows: 0–15 min, 80–20% B; 15–17 min, 5% B; 17–26 min, 80% B. For MS analysis, an Orbitrap QExactive (Thermo Fisher) was operated in polarity switching mode. To enable targeted detection of β-hydroxypyruvate and malonate, one millimolar samples were prepared and analysed in the same run as bacterial metabolite extractions. Data were analysed using the IDEOM analysis pipeline[6]. Briefly, raw LC-MS data files were converted to mzXML (Proteowizard) and separated based on positive and negative charge. Converted files were next processed with an XCMS and MzMatch based pipeline. This data was then imported into IDEOM to identify putative metabolites. Signal intensities for

annotated metabolites were statistically compared by unpaired Student's *t*-test using Prism 10 (Graphpad). Metabolites with an absolute fold change ≥ 1.5 and corrected *p*-value threshold (false discovery rate) < 0.05 were considered significant and selected for enrichment analysis. Annotated metabolite lists were separated into up- and down-regulated sets and enrichment analyses were performed using the MetaboAnalyst 6.0[7]. Significant metabolites were mapped to KEGG bacterial metabolic pathways, and over-representation analysis was conducted against the KEGG pathway library with default settings. Pathways with a corrected p-value < 0.05 were used for visualization.

Filamentous actin staining (FAS) assay

HeLa cells were seeded on sterile glass coverslips in 12 well plates at a density of 4×10^4 cells per well. MEM-HEPES with 10% fetal calf serum (FCS) and 2 mM L-glutamine was used for routine culture. The cells were incubated for 24 hours at 37°C, 5% CO_2. Overnight cultures of bacterial strains carrying pr*psm-gfp* were diluted into pre-warmed MEM-HEPES with chloramphenicol, and without L-glutamine. The strains were cultured for three hours at 37°C, 200 rpm before measuring optical density and diluting to $OD_{600\ nm}$ = 0.1. Immediately prior to infection, HeLa were washed once with PBS and fresh medium with chloramphenicol, without FCS or L-glutamine was added. Twenty microlitres of normalised bacterial suspension was added to each well (approximate multiplicity of infection, 20) and plates were centrifuged 200 × g, 2 min to synchronise infection. Infection was allowed to proceed for three hours at 37°C, 5% CO_2. The inoculum titre was checked by serial dilution and spot plating. Next, the medium was removed, and the cells washed four times with PBS. The cells were fixed by incubation in in 4% (w/v) paraformaldehyde for 15 mins before washing twice with PBS and storing at 4°C. To stain the cells, 0.1% (v/v) Triton X-100 in PBS was added and incubated for ten minutes. The cells were washed twice with PBS before incubating in a one-hundred-fold dilution of Actin Red Rhodamine Ready Probes (Thermo Fisher) in PBS for one hour. The

cells were washed twice before mounting on a drop of Slow Fade Glass with DAPI (Thermo Fisher). Images were acquired using a Leica SP8 confocal microscope.

Quantitative analysis of bacterial attachment

To enable direct quantitation of bacterial attachment to HeLa, infection experiments were performed as outlined above but using unlabelled bacterial strains. As such, chloramphenicol was not included at any stage. Following three hours of infection at an MOI of 20 as previously described, the medium was removed and retained in a separate vessel. Following each of four PBS washes, wash suspension was pooled with the medium from the same well of infected HeLa. The cells were lysed by addition of 1% (v/v) Triton X-100 in PBS. Following ten minutes of incubation, the cell layers were scratched with a pipette tip and homogenised by repeated pipetting. The lysates, wash solutions and inocula from each well were serially diluted in PBS, before spot plating to calculate the bacterial titre in each. This enabled calculation of bacterial attachment as a percentage of inoculum and bacterial growth during infection (by totalling the lysate and wash suspension counts). Data were statistically analysed in Prism 10 (Graphpad) using two-tailed Student's *t*-test.

Statistical analysis

Unless stated otherwise, experiments were performed in triplicate and analysed using statistical packages within Prism 10 (Graphpad).

Description of Supplementary Datasets

Dataset S1 – RNA-seq

Tab 1: Control vs D-serine: Read counts and EDGER generated statistical analysis of global gene expression

Tab 2: Control vs L-serine: Read counts and EDGER generated statistical analysis of global gene expression

Tab 3: Fold changes of direct NtrC regulon under D- and L-serine exposure

Tab 4: WT vs Δ*ntrC*: Read counts and EDGER generated statistical analysis of global gene expression

Tab 5: Δ*ntrC* vs Δ*ntrC* D-serine: Read counts and EDGER generated statistical analysis of global gene expression

Dataset S2 – Metabolomics IDEOM Timecourse

Tab 1: All detected peaks

Tab 2: Fold change comparison of 474 putatively identified peaks – Putative Metabolites in yellow colouring positively identified by comparison with internal standards

Dataset S3 – Metabolomics IDEOM Replicate Data

Tab 1: All detected peaks

Tab 2: Fold change comparison of 802 putatively identified peaks – Putative Metabolites in yellow colouring positively identified by comparison with internal standards

Dataset S4 – Source Data File

All quantitative data underlying main text and supplementary figures are provided

Supplementary Figures

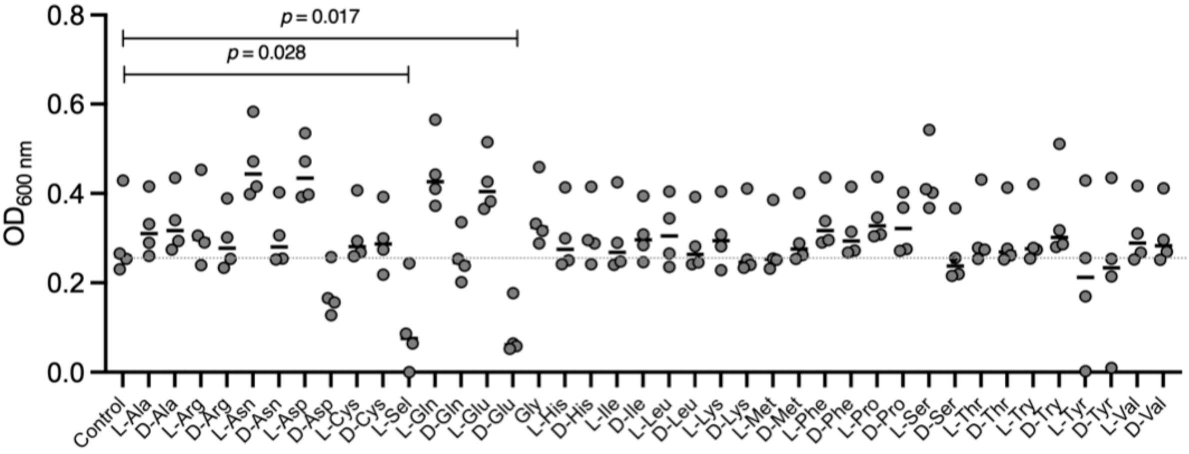

Fig. S1. L-selenocysteine and D-glutamate severely inhibit growth of EHEC in MEM-HEPES. TUV93-0 (p*LEE1-gfp+recA-rfp*) was cultured in MEM for 5 h in microtitre plates with or without inclusion of 1 mM of each indicated amino acid. Absorbance $OD_{600\ nm}$ was recorded after 5 hours using a BMG Fluostar Optima plate reader. Data are reported for 4 replicate experiments with means indicated by black lines. Statistical analysis was conducted using Ordinary One-way ANOVA with Dunnett's test for multiple comparisons. Only L-selenocysteine and D-glutamate induced significant alterations in $OD_{600\ nm}$ after 5 hours. Complete statistics for treatments found to be not significant are included in the source data. Source data are provided (Dataset S4).

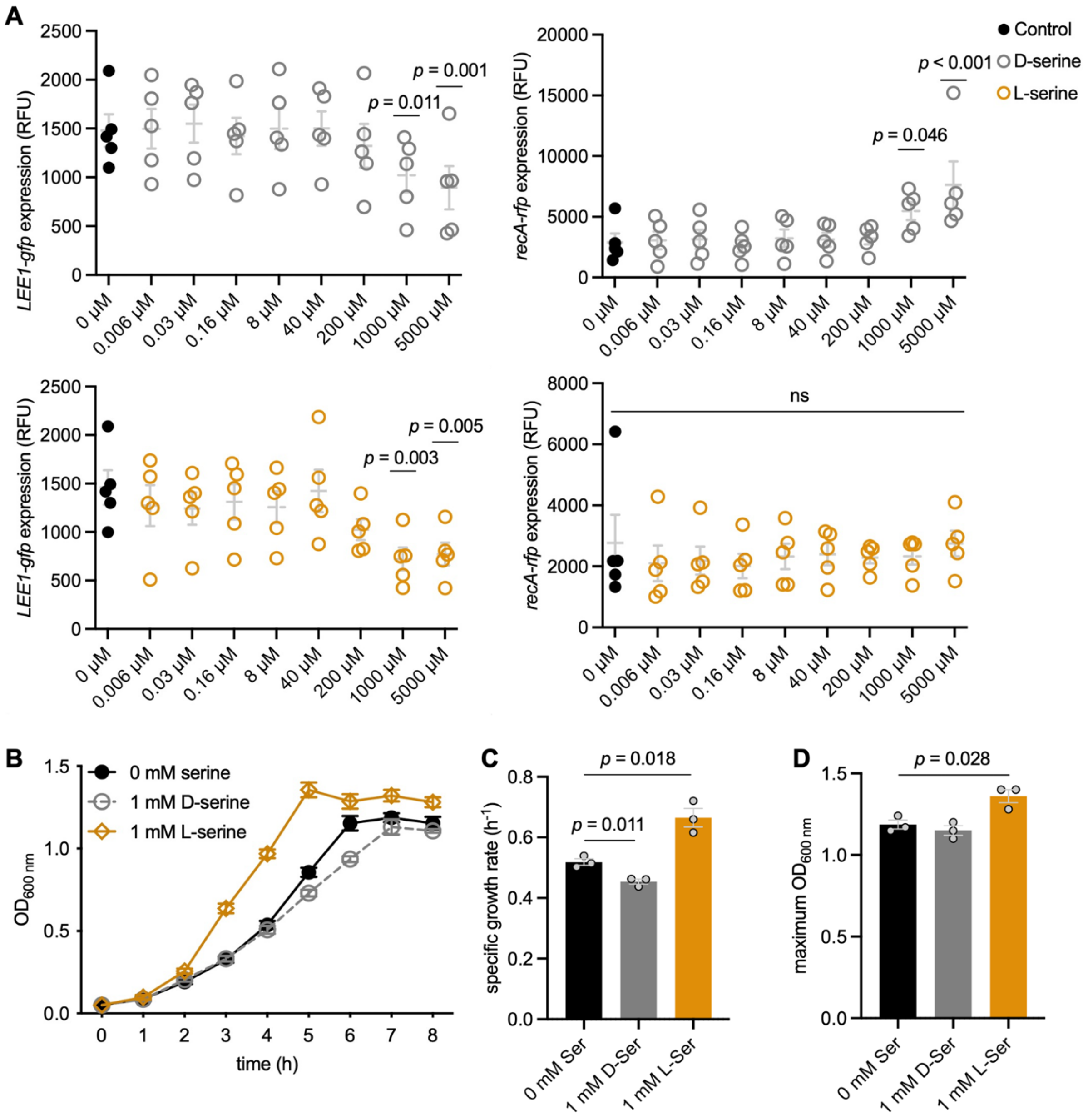

Fig. S2. Growth under conditions employed for RNA-seq, D- and L-serine dose response. (**A**) TUV93-0 (p*LEE1-gfp+recA-rfp*) was cultured in MEM for 5 h with doubling dilution series of each indicated amino acid. Fluorescence intensity (FI: $485_{ex}/520_{em}$) and OD_{600} was recorded on a Synergy HT plate reader. Normalised Relative fluorescence Units (RFU) are reported for 5 replicate experiments with standard error of the means (SEM) indicated by error bars. Statistical analysis was conducted using One way ANOVA with Dunnett's post-test. (**B**-**D**) Bacteria were cultured in MEM-HEPES in the presence and absence of 1 mM of each amino acid. Absorbance ($OD_{600\ nm}$) was recorded at hourly intervals (**B**). Specific growth rate was calculated between two and four hours using the data presented in Supplementary Fig. 2B (**C**). The maximum optical density $OD_{600\ nm}$ reached was also recorded in each replicate (**D**), indicating L-serine induced biomass increase. Statistical analysis was conducted using two-tailed Student's *t*-test. (Source data are provided (Dataset S4).

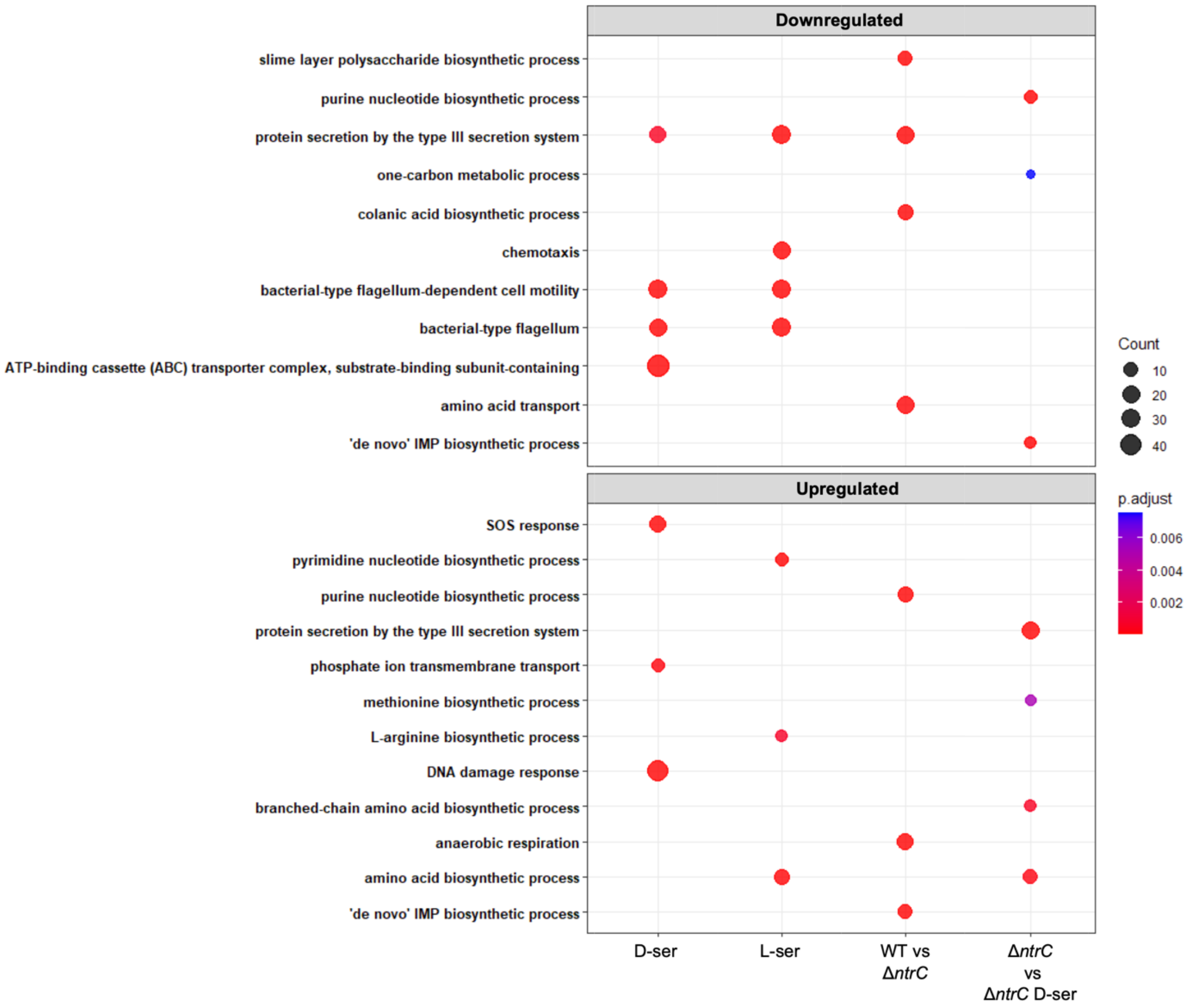

Fig. S3. Gene Ontology pathway enrichment analysis of differential expression. Over-representation analysis was conducted using the CompareCluster function in ClusterProfiler[2]. The most significantly enriched GO categories are shown for each condition. Downregulated pathways are shown in the top panel, while upregulated pathways are shown on the bottom. *p*-values were adjusted for multiple testing using the Benjamini-Hochberg method, and pathways with an adjusted *p*-value < 0.05 were considered significant.

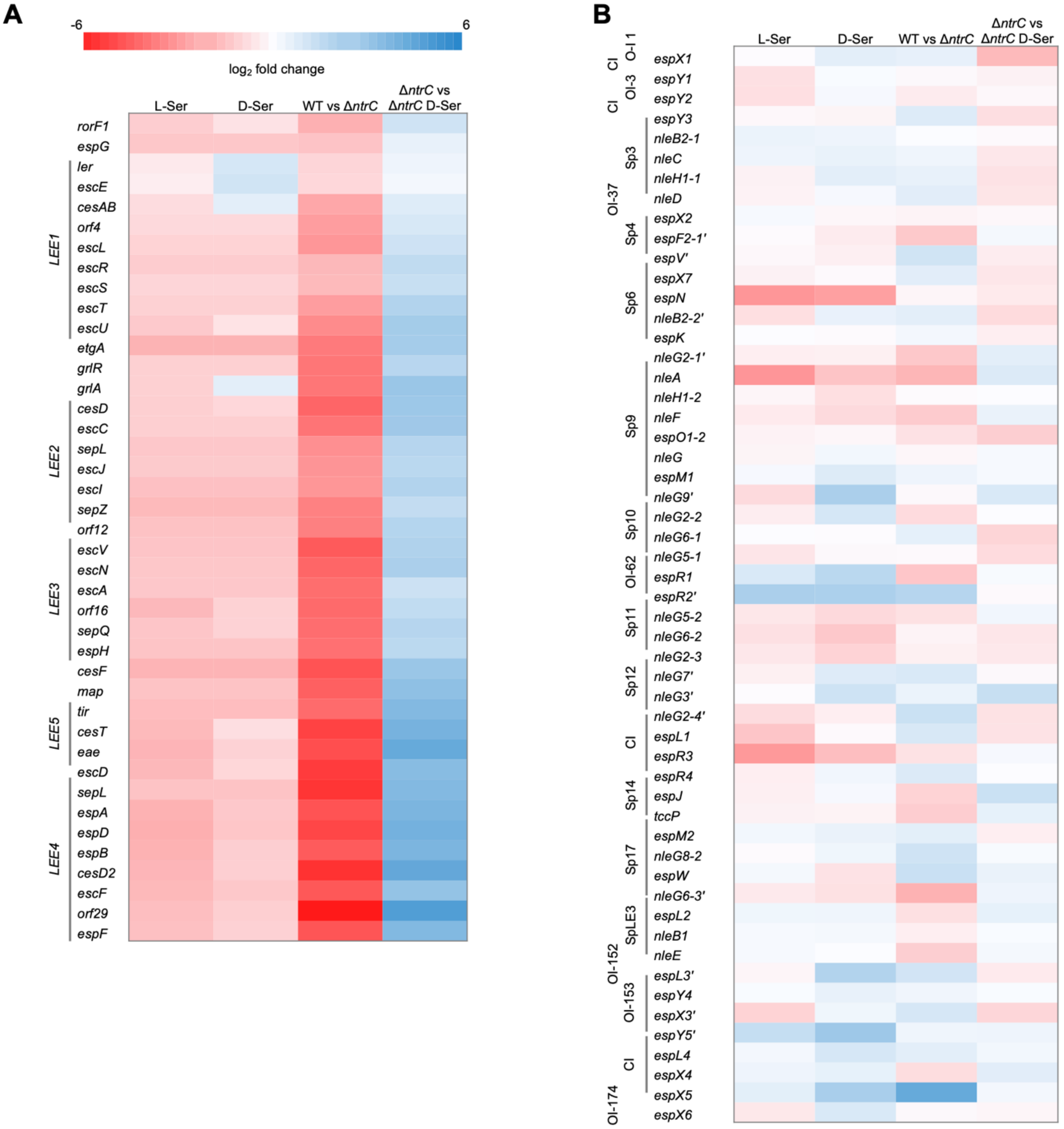

Fig. S4. Effect of L-serine, D-serine and NtrC on the locus of enterocyte effacement and non-LEE encoded effectors (NLEs). (**A** and **B**) Heatmap illustrating $\log_2$ transformed fold change data from genes encoded within the LEE (**A**) or non-LEE-encoded effector (NLE) protein encoding genes (**B**). Data were derived from RNA-seq analysis of late exponential phase cultures of the indicated strains. List of NLEs was adapted from Tobe et al. 2006[8]. OI denotes O-island; CI denotes coli island; Sp denotes lambdoid prophage encoded exchangeable effector loci.

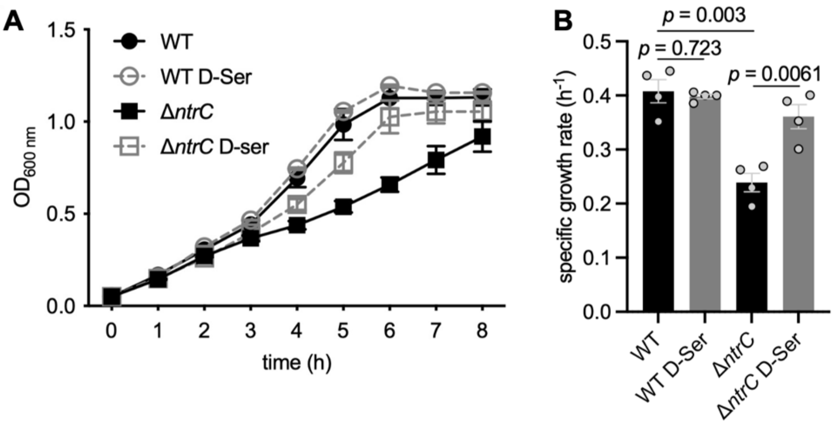

Fig. S5. Growth in MEM-HEPES is impaired by deletion of *ntrC* and can be rescued by supplementation with D-serine. TUV93-0 and the isogenic Δ*ntrC* mutant were cultured in MEM-HEPES with or without inclusion of 1 mM D-serine. Absorbance ($OD_{600\ nm}$) was recorded at hourly intervals (**A**). Specific growth rate was calculated between two and four hours using the data presented in Supplementary Fig. 4A (**B**). Statistical analysis was conducted using two-tailed Student's *t*-test. Source data are provided (Dataset S4).

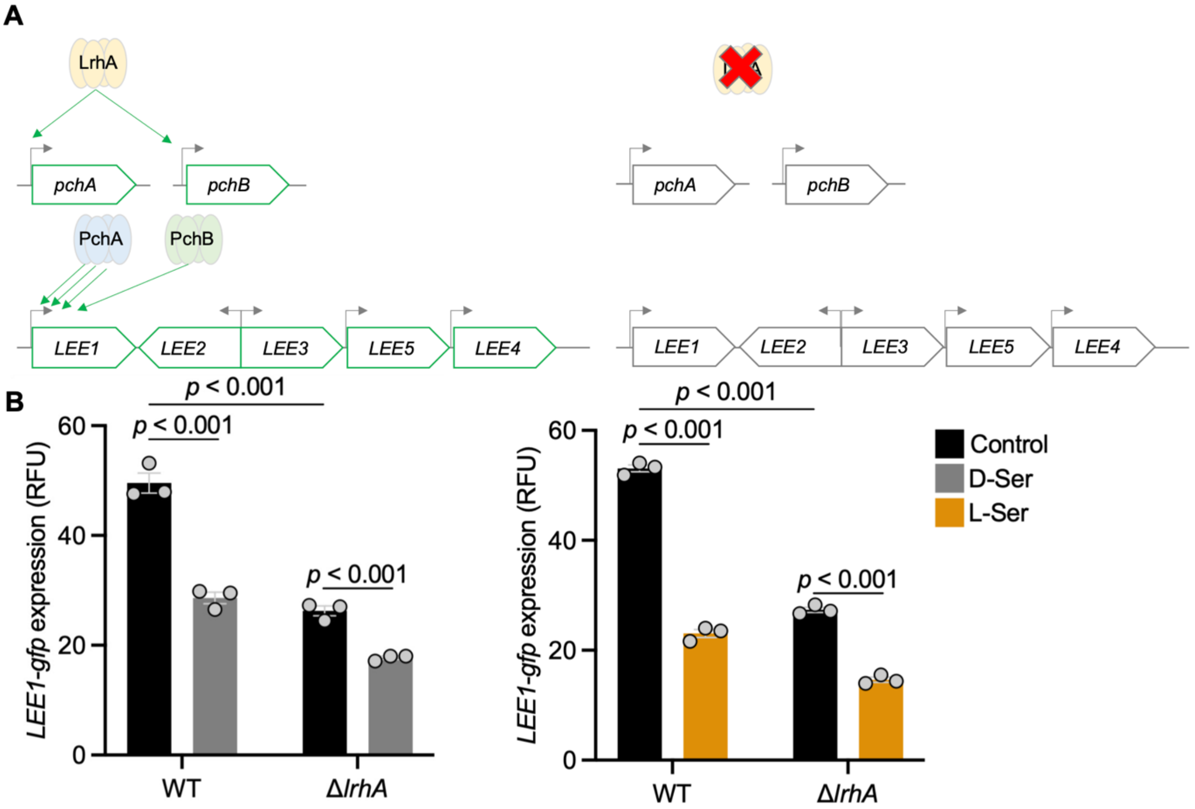

Fig. S6. Repression of the LEE type 3 secretion system is not facilitated by the LrhA-PchAB regulatory cascade. (**A**) Schematic illustration of the role of LrhA-PchAB in regulation of the LEE. Scenario to the left of panel A indicates TUV93-0 WT. LrhA is expressed in MEM-HEPES, thereby activating the known transcriptional inducers of the LEE, PchA and B. Both proteins bind to the LEE1 promoter and induce expression of the T3SS. In the Δ*lrhA* deletion mutant, pchA and pchB are not transcribed, therefore the basal level of T3SS activity is reduced. (**B**) TUV93-0 (p*LEE1-gfp+recA-rfp*) was cultured in MEM for 5 h with or without inclusion of 1 mM of each indicated amino acid. Fluorescence intensity (FI: $485_{ex}/520_{em}$ was recorded on a VarioSkan lux plate reader. Normalised Relative fluorescence Units (RFU) are reported for 3 replicate experiments with standard error of the means (SEM) indicated by error bars. Statistical analysis was conducted using two-tailed Student's *t*-test. Source data are provided (Dataset S4).

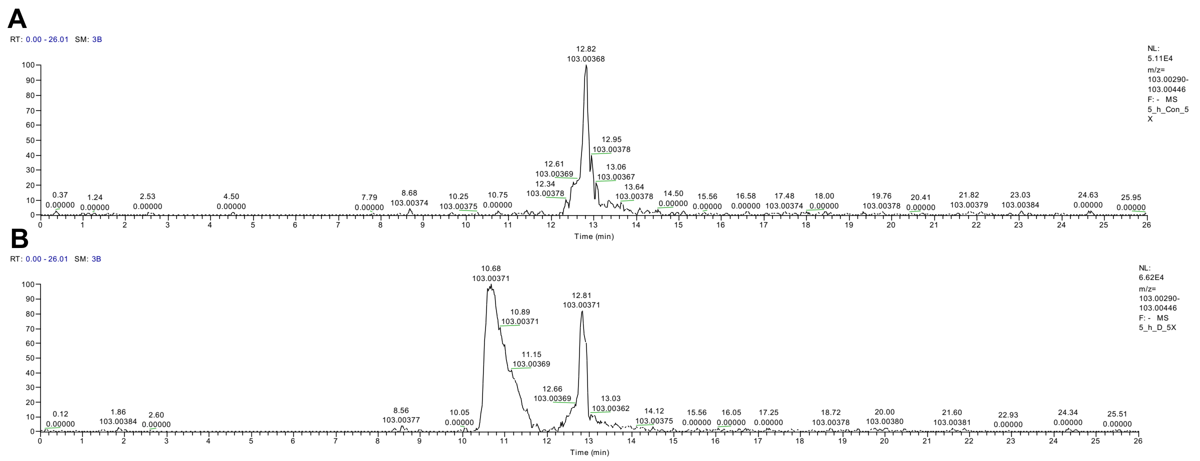

Fig. S7. Chromatograms from control cells showing-serine specific peak of m/z 103.0037 at RT = 10.68. Chromatograms displayed were derived from 5 h cultures at 5× cell density. Cells were incubated with 0mM (A) or 1 mM (B) D-serine.

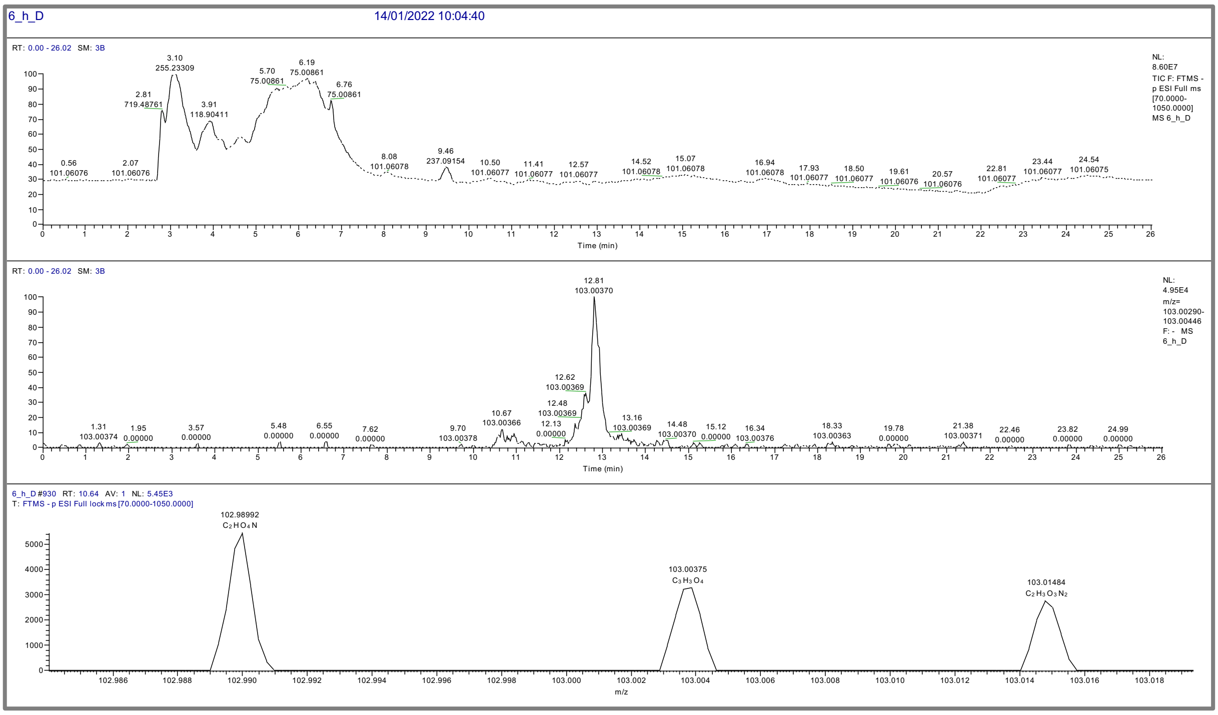

Fig. S8. Chromatograms and mass spectra from D-serine-treated cells showing m/z 103.0037 peak at RT = 10.68.

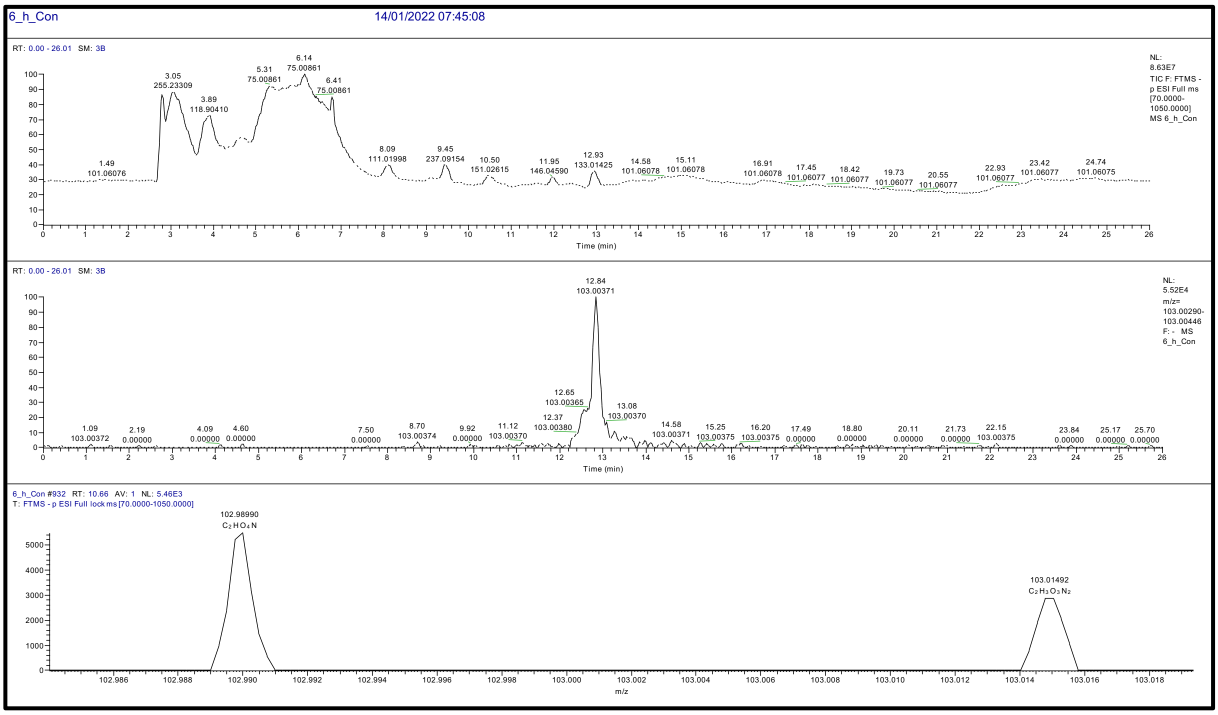

Fig. S9. Chromatograms and mass spectra from control cells showing a lack of m/z 103.0037 at RT = 10.68.

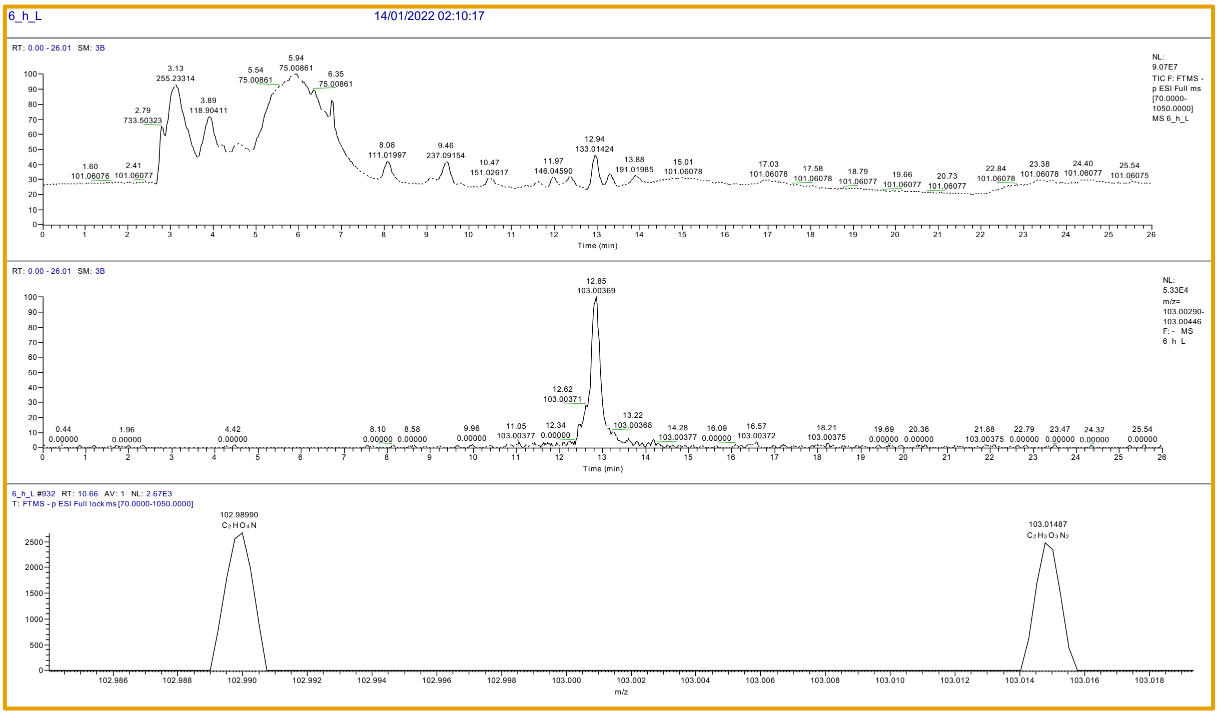

Fig. S10. Chromatograms and mass spectra from L-serine-treated cells showing a lack of m/z 103.0037 at RT = 10.68.

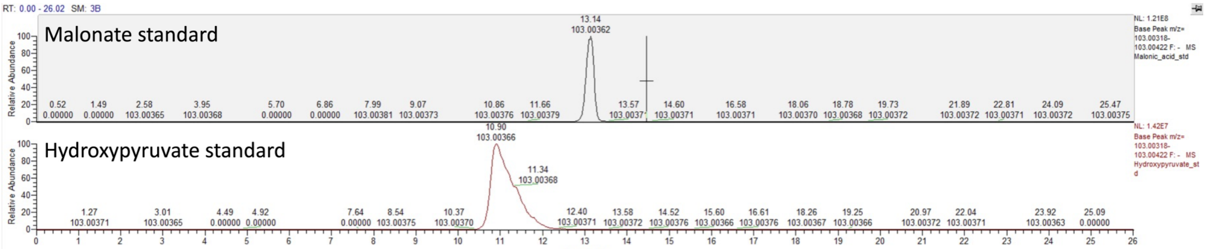

Fig. S11. Chromatograms demonstrating distinct RT for malonate and β-hydroxypyruvate.

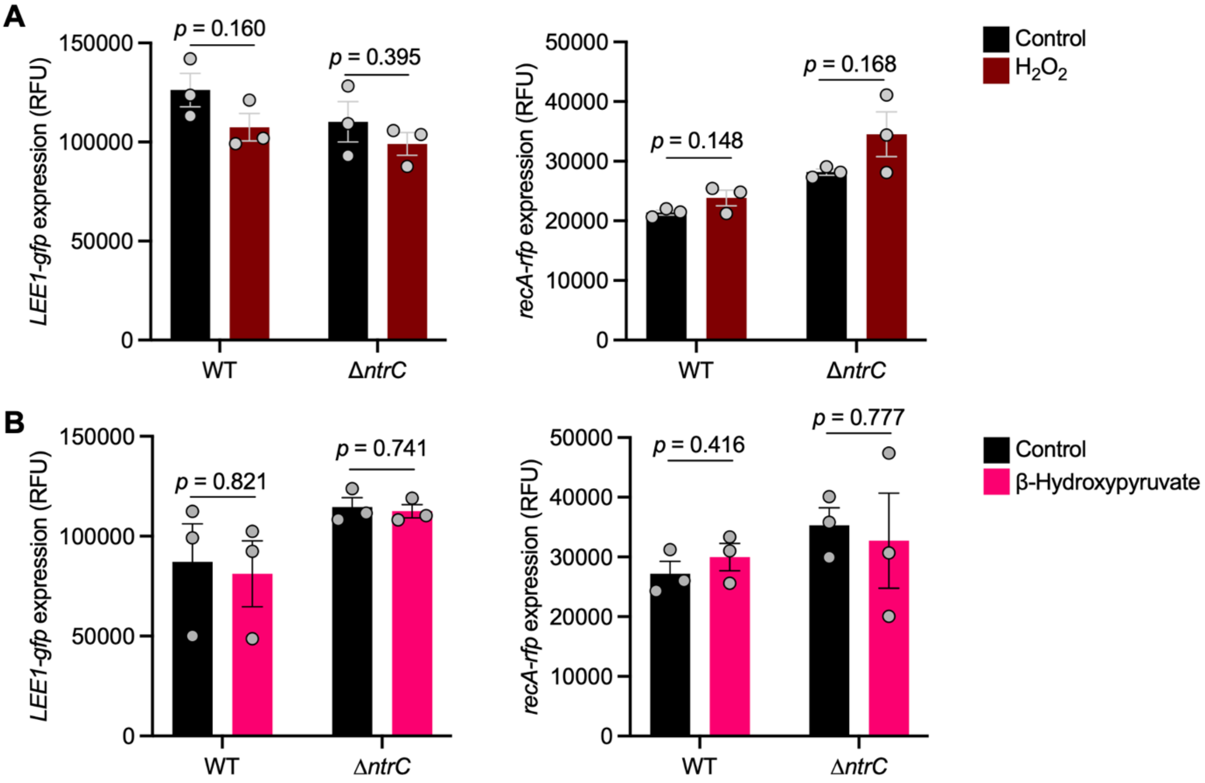

Fig. S12. Products of oxidative D-serine deamination do not modulate *LEE1p* or *recAp* activity. TUV93-0 (p*LEE1-gfp+recA-rfp*) was cultured in MEM for 5 h with 1 mM H2O2 (**A**) or 1 mM β-Hydroxypyruvate (B). Fluorescence intensity (FI: $485_{ex}/520_{em}$) and OD_{600} was recorded on a BMG Fluostar plate reader. Normalised Relative fluorescence Units (RFU) are reported for 3 replicate experiments with standard error of the means (SEM) indicated by error bars. Statistical analysis was conducted using Student's *t*-test. Source data are provided (Dataset S4).

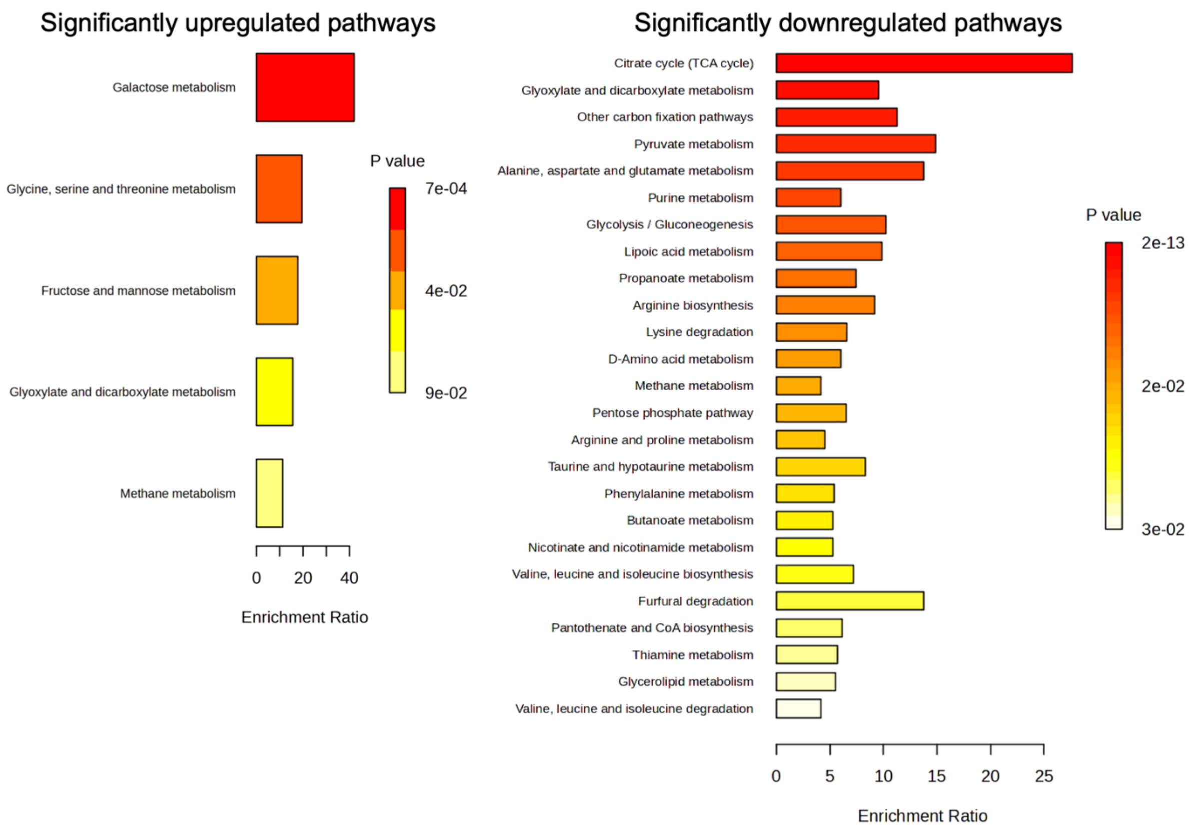

Fig. S13. MetaboAnalyst 6.0 KEGG pathway enrichment analysis (D- vs L-serine). Enrichment analysis was conducted using MetaboAnalyst 6.0[7]. Metabolites with absolute fold change ≥ 1.5 and corrected *p*-value threshold (false discovery rate-corrected) < 0.05 were mapped to KEGG bacterial metabolic pathways, and over-representation analysis was conducted against the KEGG pathway library. Pathways with a corrected p-value < 0.05 were used for visualization.

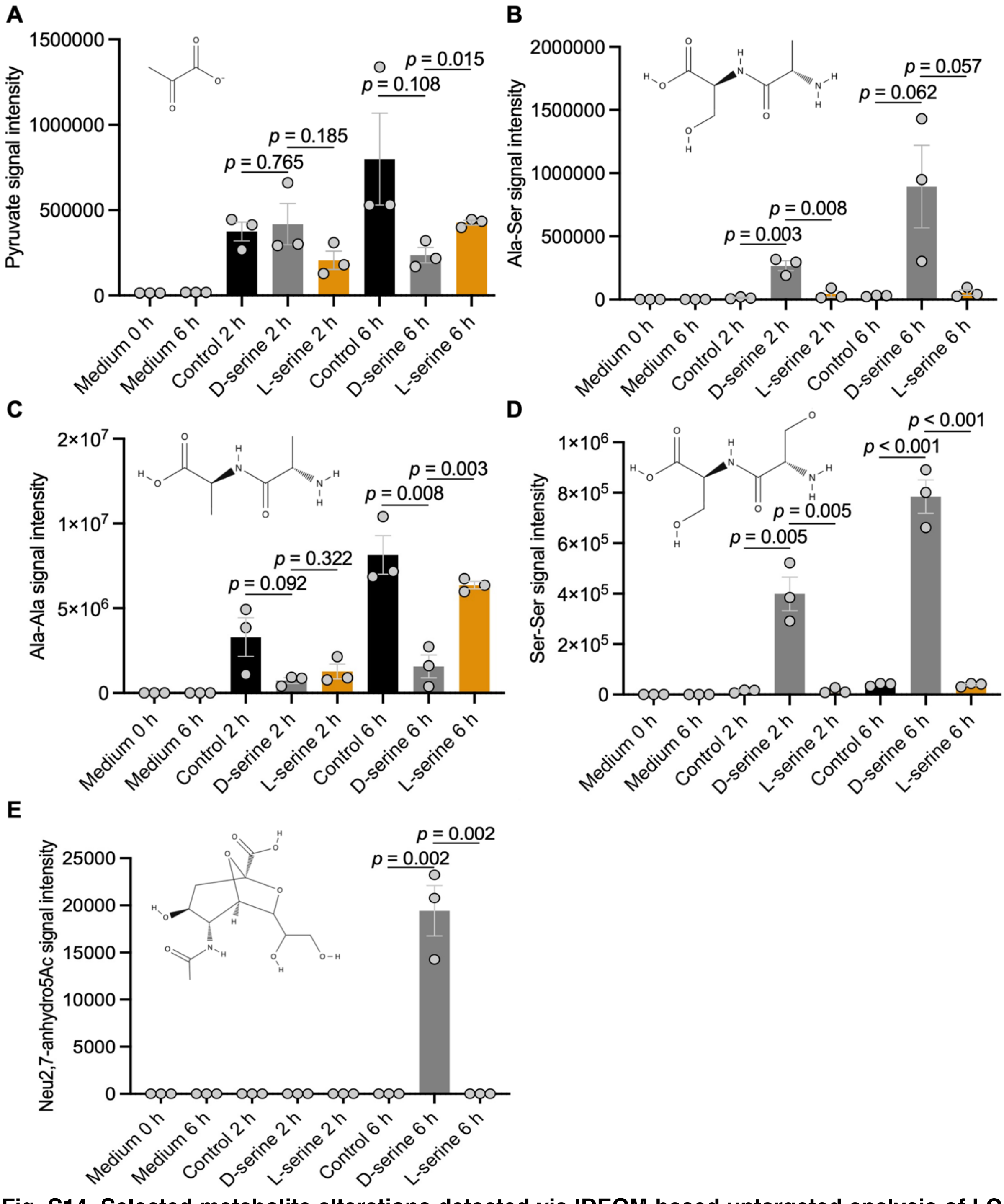

Fig. S14. Selected metabolite alterations detected via IDEOM-based untargeted analysis of LC-MS data. (**A - D**) LC-MS data were derived from TUV93-0 cultured in control MEM-HEPES, or MEM-HEPES with 1 mM of each amino acid. Metabolites include pyruvate (**A**), Ala-Ser (**B**), Ala-Ala (**C**) Ser-Ser (**D**) and 2,7 anhydro-alpha-N-acetyl neuraminic acid (**E**). Statistical analysis was conducted using two-tailed Student's *t*-test. Chemical structures (inset) were derived from Molview. Source data are provided (Dataset S4).

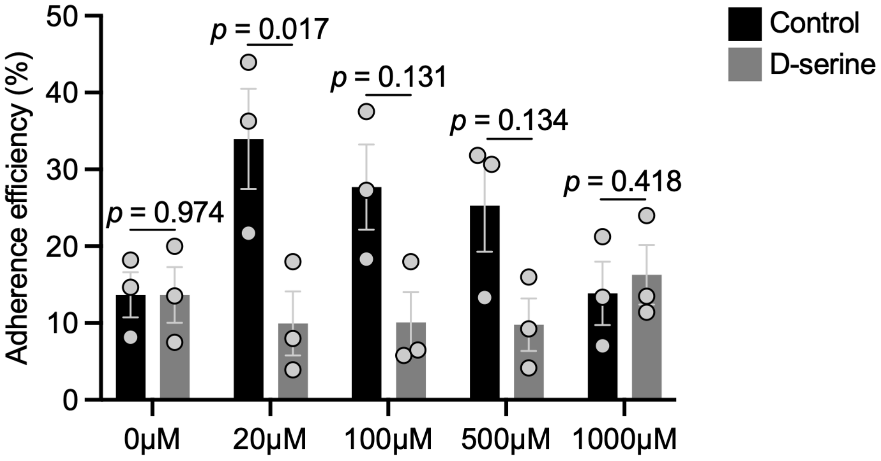

Fig. S15. Induction of *ntrC* expression by 3-Methylbenzoic acid restores the repressive effect of D-serine on adherence to HeLa cells. HeLa cells were infected with TUV93-0 Δ*ntrC* (pSS-*ntrC*) for 3 h in the presence of varying concentrations of the XylS/Pm inducer 3-Methyl benzoic acid (3-MeBzO), and in the presence and absence of 1 mM D-serine. Following four PBS washes, PBS + 1% Triton X-100 was added and incubated for fifteen minutes to lyse the HeLa cells. The cells were scraped from the plate, mixed by repeatedly pipetting and serially diluted to titre adherent bacteria. Adherent CFU is expressed as a percentage of the inoculum added. Data from 3 replicate experiments are reported with standard error of the means (SEM) indicated by error bars. Statistical analysis was conducted using two-tailed Student's *t*-test. Source data are provided (Dataset S4).

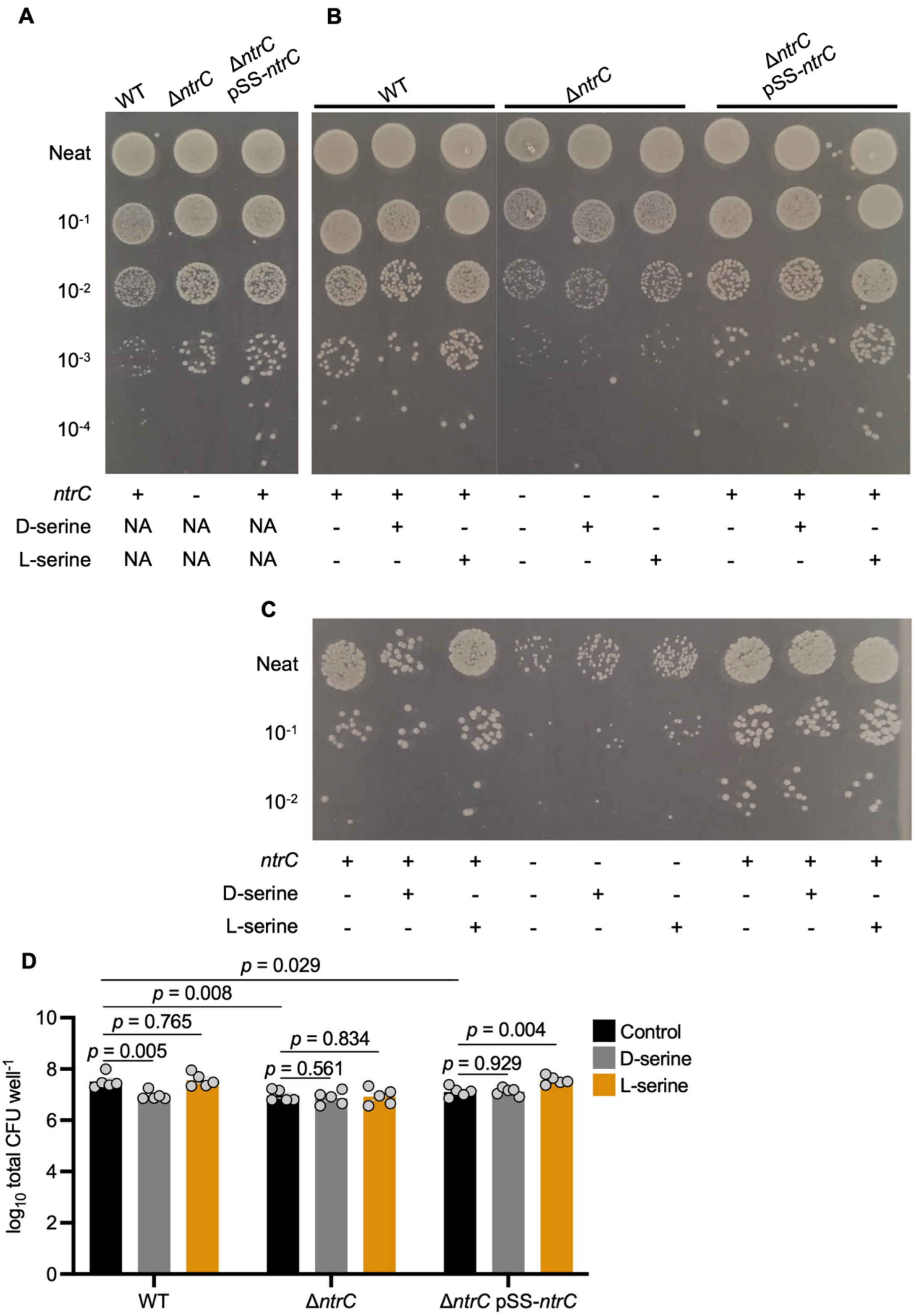

Fig. S16. Chiral enantiomers of serine display contrasting ability to interfere with attachment and growth during in vitro HeLa cell infection. (**A-C**) Bacterial titers from a representative experiment. Colony forming units were assessed in the inoculum (A), pooled wash solution and culture media (B) and lysate following washing (C). Each spot represents a five microlitre drop of the corresponding dilution. (D) Total CFU per well was calculated by adding the bacterial titre calculated from spots in panel B and panel C. Data from 5 replicate experiments are reported with standard error of the means (SEM) indicated by error bars. Data are log_{10} transformed. Statistical analysis was conducted using two-tailed Student's *t*-test. Source data are provided (Dataset S4).

Supplementary Tables

Supplementary Table 1 - Bacterial strains used in this study

Strain	Description	Reference
WT (EHEC, TUV93-0)	*E. coli* O157:H7 (Stx-negative derivative of EDL933)	[9]
E. coli DH5α	F– ϕ80*lac*ZΔM15 Δ(*lac*ZYA-*arg*F)U169 *rec*A1 *end*A1 *hsd*R17(rK–, mK+) *pho*A *sup*E44 λ–*thi*-1 *gyr*A96 *rel*A1	[10]
Δ*ntrC*	TUV93-0 carrying a deletion in *ntrC*	This study
Δ*nac*	TUV93-0 carrying a deletion in *nac*	This study
Δ*sdaA*	TUV93-0 carrying a deletion in *sdaA*	This study
Δ*sdaCB*	TUV93-0 carrying a deletion in *sdaCB*	This study
Δ*sdaCB*/*A*	TUV93-0 carrying a deletion in both *sdaCB* and *sdaA*	This study
Δ*lrhA*	TUV93-0 carrying a deletion in *lrhA*	This study
Δ*ordL*	TUV93-0 carrying a deletion in *ordL*	This study
Δ*dsdA*	TUV93-0 carrying a deletion in *dsdA*	This study
Δ*ygcU*	TUV93-0 carrying a deletion in *ygcU*	This study
Δ*dadA*	TUV93-0 carrying a deletion in *dadA*	This study
WT pDUAL	TUV93-0 with pDUAL empty reporter plasmid; chlR	[1]
WT p*LEE1p-gfp*/*recAp-rfp*	TUV93-0 with dual LEE1/recA reporter plasmid; chlR	[1]
WT p*tirp-gfp*	TUV93-0 with *tir* reporter plasmid; chlR	[11]
WT p*rpsM-gfp*	TUV93-0 with constitutive GFP expression plasmid; chlR	[11]
Δ*ntrC* pDUAL	Δ*ntrC* with pDUAL empty reporter plasmid; chlR	This study
Δ*ntrC* p*LEE1p-gfp*/*recAp-rfp*	Δ*ntrC* with dual LEE1/recA reporter plasmid; chlR	This study
Δ*ntrC* p*tirp-gfp*	Δ*ntrC* with *tir* reporter plasmid; chlR	This study
Δ*ntrC* p*rpsM-gfp*	Δ*ntrC* with constitutive GFP expression plasmid; chlR	This study
Δ*ntrC* pSS-*ntrC*	Δ*ntrC* with pSS-*ntrC* complementation plasmid; ampR	This study
Δ*ntrC* p*LEE1p-gfp*/*recAp-rfp* pSS-*ntrC*	Δ*ntrC* with dual LEE1/recA reporter plasmid and pSS-*ntrC* complementation plasmid; chlR/ampR	This study
Δ*ntrC* p*rpsM-gfp* pSS-ntrC	Δ*ntrC* with constitutive GFP expression plasmid and pSS-ntrC complementation plasmid; chlR/ampR	This study
Δ*nac* pDUAL	Δ*nac* with pDUAL empty reporter plasmid; chlR	This study
Δ*nac* p*LEE1p-gfp*/*recAp-rfp*	Δ*nac* with dual LEE1/recA reporter plasmid; chlR	This study
Δ*nac* p*tirp-gfp*	Δ*nac* with *tir* reporter plasmid; chlR	This study
Δ*na*c p*rpsM-gfp*	Δ*nac* with constitutive GFP expression plasmid; chlR	This study
Δ*sdaCB*/*A* pSS	Δ*sdaCB*/*A* with empty pSS complementation plasmid; ampR	This study
Δ*sdaCB*/*A* pSS-*sdaA*	Δ*sdaCB*/*A* with pSS-*sdaA* complementation plasmid; ampR	This study
Δ*sdaCB*/*A* pSS-*sdaB*	Δ*sdaCB*/*A* with pSS-*sdaB* complementation plasmid; ampR	This study
Δ*sdaCB*/*A* pSS-*sdaC*	Δ*sdaCB*/*A* with pSS-*sdaC* complementation plasmid; ampR	This study
Δ*sdaCB*/*A* pSS-*sdaCB*	Δ*sdaCB*/*A* with pSS-*sdaCB* complementation plasmid; ampR	This study
Δ*lrhA* pDUAL	Δ*lrhA* with pDUAL empty reporter plasmid; chlR	This study

Δ*lrhA* p*LEE1p-gfp*/*recAp-rfp*	Δ*lrhA* with dual LEE1/recA reporter plasmid; chlR	This study
Δ*dsdA* pDUAL	Δ*dsdA* with pDUAL empty reporter plasmid; chlR	This study
Δ*dsdA* p*LEE1p-gfp*/*recAp-rfp*	Δ*dsdA* with dual LEE1/recA reporter plasmid; chlR	This study
Δ*ordL* pDUAL	Δ*ordL* with pDUAL empty reporter plasmid; chlR	This study
Δ*ordL* p*LEE1p-gfp*/*recAp-rfp*	Δ*ordL* with dual LEE1/recA reporter plasmid; chlR	This study
Δ*ygcU* pDUAL	Δ*ygcU* with pDUAL empty reporter plasmid; chlR	This study
Δ*ygcU* p*LEE1p-gfp*/*recAp-rfp*	Δ*ygcU* with dual LEE1/recA reporter plasmid; chlR	This study
Δ*dadA* pDUAL	Δ*dadA* with pDUAL empty reporter plasmid; chlR	This study
Δ*dadA* p*LEE1p-gfp*/*recAp-rfp*	Δ*dadA* with dual LEE1/recA reporter plasmid; chlR	This study

Supplementary Table 2 - Plasmids used in this study

Plasmid	Description	Reference
pSIM18	Lambda red helper plasmid; 28°C temperature sensitive origin, 42°C inducible red recombinase system; hygR	[12]
pKD3	Lambda red PCR template, R6Kɣ origin, chlR flanked by FRT sites for removal	[3]
pCP20	FRT recombinase plasmid for excision of chlR from mutants, ampR	[3]
p*rpsM-gfp*	Constitutive *gfp* expression plasmid, chlR	[11]
pDUAL	Promoterless *gfp* and *rfp* reporter plasmid; chlR	[1]
p*LEE1p-gfp+recAp-rfp*	Dual TUV93-0 *LEE1p* and *recAp* reporter plasmid; chlR	[1]
p*tirp-gfp*	TUV93-0 *LEErp* (*tirP*) reporter plasmid; chlR	[11]
pSS	Synthetic construct derived from pBR322 and pSEVA238 for benzoic acid inducible XylS/Pm expression; ampR	This study
pSS-*ntrC*	pSS plasmid with *ntrC* cloned downstream of XylS/Pm promoter; ampR	This study
pSS-*sdaA*	pSS plasmid with *sdaA* cloned downstream of XylS/Pm promoter; ampR	This study
pSS-*sdaB*	pSS plasmid with *sdaB* cloned downstream of XylS/Pm promoter; ampR	This study
pSS-*sdaC*	pSS plasmid with *sdaC* cloned downstream of XylS/Pm promoter; ampR	This study
pSS-*sdaCB*	pSS plasmid with *sdaCB* cloned downstream of XylS/Pm promoter; ampR	This study

ampR: ampicillin resistance; chlR: chloramphenicol resistance; hygR: hygromycin resistance;

Supplementary Table 3 - Oligonucleotides used in this study

Oligo	Sequence (5'-3')	Use
ntrC-red-F	CATACCGAGTTCTCGGTTTACCTGCCTATCAGGAAATAAAG GTGACGTTTGTGTAGGCTGGAGCTGCTTC	Deletion of *ntrC*
ntrC-red-R	AATACCAGCAATTTGCGCTCAATAATCAATCTTTACACACAA GCCGTGAACATATGAATATCCTCCTTAG	Deletion of *ntrC*
ntrC.check.R	CGGAAGGCGGTGAAATCATT	Check for *ntr*C deletion
ntrC.184.R	caatccatgccaacccgttcCGTTTTCCGGGCAAGATCAT	Check for *ntr*C deletion
Nac-DelA-184	gcacctgaagtcagccccatacgatCCTGTGCAAATTAGTTCTGGTTC	Deletion of *nac* upstream (AB) product
Nac-DelB-pKD	acttcgaagcagctccagcctacacGTTCATGTTGCCTCCGGTTT	Deletion of *nac* upstream (AB) product
Nac-DelC-pKD	aggaactaaggaggatattcatatgCAGTGGCAATTGGTGAGCTA	Deletion of *nac* downstream (CD) product
Nac-DelD-184	gcctacaatccatgccaacccgttcTACTCAAACGCTCGCTGGC	Deletion of *nac* downstream (CD) product
pkD-F	GTGTAGGCTGGAGCTGCTTC	Amplification of pKD3 *cat* cassette
pKD-R	CATATGAATATCCTCCTTAG	Amplification of pKD3 *cat* cassette
Nac-DelABCD-check-F	GGATGGTTCAAAGTGGCTGG	Check for *nac* deletion WT = 2159; Insertion = 2282; Clean = 1352
Nac-DelABCD-check-R	AGCTGCGGGTCATTACTCAA	Check for *nac* deletion WT = 2159; Insertion = 2282; Clean = 1352
pACYC.lin.Fw	GAACGGGTTGGCATGGATTG	Linearisation of pACYC184, excision of tetR, product: 3052
pACYC.lin.Rev	ATCGTATGGGGCTGACTTCA	Linearisation of pACYC184, excision of tetR, product: 3052
pACYC-seq-For2	CAAGAGATTACGCGCAGACC	Check for inserts in pACYC184 assemblies
pACYC.check.R	GCATTCACAGTTCTCCGCAA	Check for inserts in pACYC184 assemblies
sdaA.red.F	TGTTATTAGTTCGTTACTGGAAGTCCAGTCACCTTGTCAGGA GTATTATCGTGTAGGCTGGAGCTGCTTC	Deletion of *sdaA*
sdaA.red.R	AAAGCGGGGATAAATTCGCCCATCCGTTGCAGATGGGCGA GTAAGAAGTACATATGAATATCCTCCTTAG	Deletion of *sdaA*
sdaCB.red.F	GGCTGAACTGGCTAAAAGCTGAATTATTTGCATTCCTCCAG GAGAAATAGGTGTAGGCTGGAGCTGCTTC	Deletion of *sdaCB*
sdaCB.red.R	GGATGAGAAATCGGGAAGAGGCCTCGCAAAACGAGGCCTC TGGAGAGCGACATATGAATATCCTCCTTAG	Deletion of *sdaCB*
sdaA.184.F	tgaagtcagccccatacgatAGGCATTACATCTGGGTCGT	Check for *sdaA* deletion
sdaA.184.R	caatccatgccaacccgttcAATAAATCGCACGCTCAGGG	Check for *sdaA* deletion
sdaCB.184.F	tgaagtcagccccatacgatCGGAAGAAGTCAGATGTCGT	Check for *sdaCB* deletion
sdaCB.184.R	caatccatgccaacccgttcTGGCTGTGCATAATGAGCTG	Check for *sdaCB* deletion
pSS-lin-F:	AACCCGGGGATCCTCTAG	Linearisation of pSS
pSS-lin-R:	TACCGAGCTTTCTCCTCTTTAATTC	Linearisation of pSS
pSS-check-F	gcagtgtccggtttgatagg	Check for insert assembly in pSS, Empty = 267 bp
pSS-check-R	gactagtcgccagggttttc	Check for insert assembly in pSS, Empty = 267 bp
sdaA-pSS-F	gaattaaagaggagaaagctcggtaGTGATTAGTCTATTCGACATGTT TAAGG	Amplification of *sdaA* for assembly in pSS
sdaA-pSS-R	gtcgactctagaggatccccgggttATGCGGGCCACTCTGTAAG	Amplification of *sdaA* for assembly in pSS
sdaC-pSS-F	gaattaaagaggagaaagctcggtaATGGAAACGACTCAAACC	Amplification of *sdaC* for assembly in pSS

sdaC-pSS-R	gtcgactctagaggatccccgggttCGCGAAAGGACTTAGCTG	Amplification of *sdaC* for assembly in pSS
sdaB-pSS-F	gaattaaagaggagaaagctcggtaATGATTAGCGTATTCGATATTTTCAAAATC	Amplification of *sdaB* for assembly in pSS
sdaB-pSS-R	gtcgactctagaggatccccgggttAGAAATCGGGAAGAGGCCTC	Amplification of *sdaB* for assembly in pSS
lrhA-DelA-184	gcacctgaagtcagccccatacgatGCGGAATTAATGAATGAGGATGA	Deletion of *lrhA* upstream (AB) product
lrhA-DelB-pKD	acttcgaagcagctccagcctacacCGGACGATTTGCACTTATCAT	Deletion of *lrhA* upstream (AB) product
lrhA-DelC-pKD	aggaactaaggaggatattcatatgCCGGAAGGGGATGATTCATTG	Deletion of *lrhA* downstream (CD) product
lrhA-DelD-184	gcctacaatccatgccaacccgttcGGACGCCACATCGATTCATG	Deletion of *lrhA* downstream (CD) product
lrhA-check-F	GAAACGCGATGATGATATTTCCG	Check for *lrhA* deletion
lrhA-check-R	ACTCATCAAAAGTAGCGCCAG	Check for *lrhA* deletion
ordL.red.F	tgaaaaattcaccgaactgaaaaccatctggataagcctggaggcctgaaGTGTAGGCTGGAGCTGCTTC	Deletion of *ordL*
ordL.red.R	cattgttgctcatgtttcacaccttttgactcttcactgttaatcggaaaCATATGAATATCCTCCTTAG	Deletion of *ordL*
ordL.check.F	GTCACGCGTTTCACATCAGA	Check for *ordL* deletion
ordL.check.R	CTCAACATCCTTCAGCGTGG	Check for *ordL* deletion
dsdA.red.F	gcgggcacattcctgctgtcatttatcatctaagcgcaaagagacgtactGTGTAGGCTGGAGCTGCTTC	Deletion of *dsdA*
dsdA.red.R	gcagcatcgctcacccagggaaaggattgcgatgctgcgttgaaacgttaCATATGAATATCCTCCTTAG	Deletion of *dsdA*
dsdA.check.F	GATTGCTATCGGTTCAGGCG	Check for *dsdA* deletion
dsdA.check.R	GGTTTTGGTTTGGTTTGCGAAA	Check for *dsdA* deletion
ygcU.red.F	acatcatcatcggcaacgaaggtgcattgtgctatatcactgaagtaacaGTGTAGGCTGGAGCTGCTTC	Deletion of *ygcU*
ygcU.red.R	gagaagccgaaaaggcaacgccccatcatcgcggaacgctgcctgacacaCATATGAATATCCTCCTTAG	Deletion of *ygcU*
ygcU.check.F	TCGAAGATATGGTCGTTGGTCT	Check for *ygcU* deletion
ygcU.check.R	CGCAGCAATTCGACAGTGAA	Check for *ygcU* deletion
dadA.red.F	tgtaattagattattattcttttactgtatctaccgttatcggagtggctGTGTAGGCTGGAGCTGCTTC	Deletion of *dadA*
dadA.red.R	aatgcctgcagatcgaggctggcctgtatcggacgggtcatctcgtttccCATATGAATATCCTCCTTAG	Deletion of *dadA*
dadA.check.F	TAAAGACGCCATATTGCCGC	Check for *dadA* deletion
dadA.check.R	AATGCAAAGCCATCGGTAGC	Check for *dadA* deletion

Supplementary References

1. O'Boyle, N., Connolly, J. P., Tucker, N. P. & Roe, A. J. Genomic plasticity of pathogenic *Escherichia coli* mediates D-serine tolerance via multiple adaptive mechanisms. *Proc. Natl. Acad. Sci.* **117**, 22484–22493 (2020).
2. Yu, G., Wang, L.-G., Han, Y. & He, Q.-Y. clusterProfiler: an R package for comparing biological themes among gene clusters. *Omics J. Integr. Biol.* **16**, 284–287 (2012).
3. Datsenko, K. A. & Wanner, B. L. One-step inactivation of chromosomal genes in *Escherichia coli* K-12 using PCR products. *Proc. Natl. Acad. Sci.* **97**, 6640–6645 (2000).
4. Calles, B., Goñi-Moreno, Á. & de Lorenzo, V. Digitalizing heterologous gene expression in Gram-negative bacteria with a portable ON/OFF module. *Mol. Syst. Biol.* **15**, e8777 (2019).
5. Balbás, P. *et al.* Plasmid vector pBR322 and its special-purpose derivatives — a review. *Gene* **50**, 3–40 (1986).
6. Creek, D. J., Jankevics, A., Burgess, K. E. V., Breitling, R. & Barrett, M. P. IDEOM: an Excel interface for analysis of LC–MS-based metabolomics data. *Bioinformatics* **28**, 1048–1049 (2012).
7. Pang, Z. *et al.* MetaboAnalyst 6.0: towards a unified platform for metabolomics data processing, analysis and interpretation. *Nucleic Acids Res.* **52**, W398–W406 (2024).
8. Tobe, T. *et al.* An extensive repertoire of type III secretion effectors in *Escherichia coli* O157 and the role of lambdoid phages in their dissemination. *Proc. Natl. Acad. Sci.* **103**, 14941–14946 (2006).
9. Campellone, K. G., Giese, N., Tipper, O. J. & Leong, J. M. A tyrosine-phosphorylated 12-amino-acid sequence of enteropathogenic *Escherichia coli* Tir binds the host adaptor protein Nck and is required for Nck localization to actin pedestals. *Mol. Microbiol.* **43**, 1227–1241 (2002).

10. Hanahan, D. Studies on transformation of *Escherichia coli* with plasmids. *J. Mol. Biol.* **166**, 557–580 (1983).

11. Roe, A. J. *et al.* Co-ordinate single-cell expression of LEE4- and LEE5-encoded proteins of *Escherichia coli* O157:H7. *Mol. Microbiol.* **54**, 337–352 (2004).

12. Chan, W. *et al.* A recombineering based approach for high-throughput conditional knockout targeting vector construction. *Nucleic Acids Res.* **35**, e64–e64 (2007).
